# Microstructure predicts non-motor outcomes following deep brain stimulation in Parkinson’s disease

**DOI:** 10.1038/s41531-024-00717-y

**Published:** 2024-05-18

**Authors:** Philipp A. Loehrer, Miriam H. A. Bopp, Haidar S. Dafsari, Sieglinde Seltenreich, Susanne Knake, Christopher Nimsky, Lars Timmermann, David J. Pedrosa, Marcus Belke

**Affiliations:** 1 Department of Neurology, Philipps-University Marburg, Marburg, Germany; 2 MRC Brain Network Dynamics Unit, Nuffield Department of Clinical Neurosciences, University of Oxford, Oxford, United Kingdom; 3 Department of Neurosurgery, Philipps-University Marburg, Marburg, Germany; 4 Center for Mind, Brain and Behavior (CMBB), Marburg, Germany; 5 Department of Neurology, University Hospital Cologne, Cologne, Germany; 6 Center for Personalized Translational Epilepsy Research (CePTER) Consortium, Cologne, Germany

**Keywords:** Parkinson's disease, Predictive markers, Parkinson's disease, Outcomes research

## Abstract

Deep brain stimulation of the subthalamic nucleus (STN-DBS) effectively treats motor and non-motor symptoms in advanced Parkinson’s disease (PD). As considerable interindividual variability of outcomes exists, neuroimaging-based biomarkers, including microstructural metrics, have been proposed to anticipate treatment response. In this prospective open-label study, we sought to detect microstructural properties of brain areas associated with short-term non-motor outcomes following STN-DBS. Thirty-seven PD patients underwent diffusion MRI and clinical assessments at preoperative baseline and 6-month follow-up. Whole brain voxel-wise analysis assessed associations between microstructural metrics and non-motor outcomes. Intact microstructure within specific areas, including the right insular cortex, right putamen, right cingulum, and bilateral corticospinal tract were associated with greater postoperative improvement of non-motor symptom burden. Furthermore, microstructural properties of distinct brain regions were associated with postoperative changes in sleep, attention/memory, urinary symptoms, and apathy. In conclusion, diffusion MRI could support preoperative patient counselling by identifying patients with above- or below-average non-motor responses.

## Introduction

Deep brain stimulation (DBS) of the subthalamic nucleus (STN) is an established treatment for advanced Parkinson’s disease (PD), improving motor- and non-motor symptoms^1–4^. Despite significant therapeutic benefits at the group level, considerable variability in individual outcomes exists^5^. To predict postoperative outcomes and thereby improve preoperative patient counselling, the use of neuroimaging-based markers has been proposed^5^. In this regard, quantitative MRI techniques such as neurite orientation dispersion and density imaging (NODDI) have lately gained increasing attention to track disease progression and treatment response^6^. NODDI is a multi-compartmental diffusion-weighted MRI technique, which facilitates the assessment of specific microstructural properties directly related to neurite morphology^7^. The model provides two voxelwise metrics of neurite morphology: the neurite density index (NDI), describing the density of axons and dendrites within a voxel, and the neurite orientation dispersion index (ODI), characterizing the variability of neurite orientations, i.e. how parallel they are. Importantly, the relationship between NODDI metrics and underlying tissue properties has recently been confirmed histologically^8^. Despite its sensitivity and specificity, NODDI protocols have a clinically feasible data acquisition time, making them an important tool for use in clinical research^6^. Previous studies employing NODDI in PD have shown that the model is capable of differentiating PD patients from healthy controls^9^ and patients with atypical Parkinsonism^10^. Furthermore, NODDI characterized disease-related pathology, such as retrograde degeneration of the nigrostriatal pathway^11^, and its metrics were associated with bimanual motor control^12^, disease severity, and duration^6,9^. Combining NODDI with conventional diffusion tensor imaging (DTI) metrics such as fractional anisotropy in a multi-parametric analysis therefore provides complementary information on microstructural properties and may serve as an imaging-based marker to facilitate treatment prediction and inform patient counselling. Thus, we sought to demonstrate that a multi-parametric quantitative MRI approach can detect microstructural properties of brain areas that predict non-motor outcomes following STN-DBS in PD. Specifically, we aimed to identify regions whose microstructural metrics were associated with (1) changes in overall non-motor symptom burden, (2) changes in non-motor symptom domains, showing significant improvements after STN-DBS, and (3) changes in neuropsychiatric symptoms such as apathy. The results of the present study should help to guide preoperative patient counselling by identifying microstructure that predicts above- or below-average non-motor response to STN-DBS.

## Results

### Clinical outcomes

Longitudinal changes of clinical outcomes are reported in Table 1. At the 6-month follow-up, we observed improvements of Non-Motor Symptom Scale-total score (NMSS-T), (*p* = 0.007, r_es_ = −0.34), Parkinson’s Disease Questionnaire (PDQ)-8 SI (*p* < 0.001, Cohen’s d: 0.67), and Scales for Outcomes in PD-Motor Function (SCOPA-M) total score (*p* < 0.001, r_es_ = −0.47). Furthermore, reductions of the levodopa equivalent daily dose (LEDD) (*p* < 0.001, Cohen’s d: 1.2) and LEDD of dopamine agonists (LEDD-DA; *p* < 0.001, Cohen’s d: 0.7) were observed. No changes in Apathy Evaluation Scale (AES) were observed (*p* = 0.71, Cohen’s d: 0.06). Analysis of NMSS domains revealed beneficial effects of STN-DBS on sleep/fatigue (domain 2; *p* < 0.001, r_es_ = −0.44), attention/memory (domain 5; *p* = 0.024, r_es_ = −0.29), and urinary symptoms (domain 7; *p* = 0.028, r_es_ = −0.28). Analysis of SCOPA-M domains showed improvements in motor examination (*p* = 0.008, r_es_ = −0.33), activities of daily living *(p* = 0.003, r_es_ = −0.38), and motor complications (*p* < 0.001, r_es_ = −0.44) at the 6-month follow-up. Results of the correlation analysis between change scores of clinical data and NMSS-T are reported in the supplementary material (Supplementary Table 1).

**Table 1:** Baseline characteristics and outcomes at baseline and 6-month follow-up

	*n*	*M*	*SD*					
Age [y]	37	58.8	7.3					
Disease duration [y]	37	8.9	4.3					
Sex (female/male) [%]	37	9/28	[24.3/75.7]					

	Baseline			6-MFU			Baseline vs. 6-MFU	
	*n*	*M*	*SD*	*n*	*M*	*SD*	*p*	*effect size*
NMSS total score	37	71.9	39.1	37	48.4	29.1	**0.007**	**−0.34**
Cardiovascular	37	1.6	2.7	37	1.1	2.5	0.238	−0.15
Sleep / fatigue	37	16.9	11.5	37	7.9	7.6	**<0.001**	**−0.44**
Mood / apathy	37	11.6	13.3	37	6.0	7.6	0.115	−0.21
Perceptual problems/ hallucinations	37	0.9	2.5	37	1.1	3.0	0.865	−0.03
Attention / memory	37	7.0	6.5	37	5.0	5.9	**0.024**	**−0.29**
Gastrointestinal	37	5.6	5.0	37	4.4	4.9	0.237	−0.16
Urinary	37	13.2	10.4	37	9.1	10.2	**0.028**	**−0.28**
Sexual function	37	3.8	5.0	37	3.0	4.3	0.313	−0.13
Miscellaneous	37	11.2	8.9	37	10.7	7.8	0.865	−0.02
PDQ-8 SI	37	32.3	14.8	37	22.6	14.1	**<0.001**	**0.67**
SCOPA-M total score	37	19.8	6.2	37	12.9	6.4	**<0.001**	**−0.47**
SCOPA-M-motor examination	37	9.4	4.2	37	6.7	4.1	**0.008**	**−0.33**
SCOPA-M-activities of daily living	37	6.6	2.7	37	4.1	3.1	**0.003**	**−0.38**
SCOPA-M-motor complications	37	3.8	2.3	37	2.1	2.4	**<0.001**	**−0.44**
LEDD [mg]	37	962.3	393.2	37	537.6	269.0	**<0.001**	**1.2**
LEDD DA [mg]	37	261.8	133.6	37	170.6	123.8	**<0.001**	**0.7**
AES	37	32.7	9.9	37	32.2	7.0	**0.715**	**0.06**

Demographic characteristics and outcome parameters at baseline and 6-months follow-up. Reported *p* values are corrected for multiple comparisons using Benjamini-Hochberg’s method. Bold font highlights significant results, *p* < 0.05.

*6-MFU* 6-month follow-up, *AES* Apathy Evaluation Scale, *LEDD* Levodopa equivalent daily dose, *LEDD-DA* LEDD of Dopamine Agonists, *NMSS* Non-Motor Symptom Scale, *PDQ-8* SI 8-item Parkinson’s Disease Questionnaire summary index, *SCOPA* Scales for Outcomes in Parkinson’s disease.

### Interaction between Fractional Anisotropy and postoperative nonmotor symptom change

**Fig. 1:**
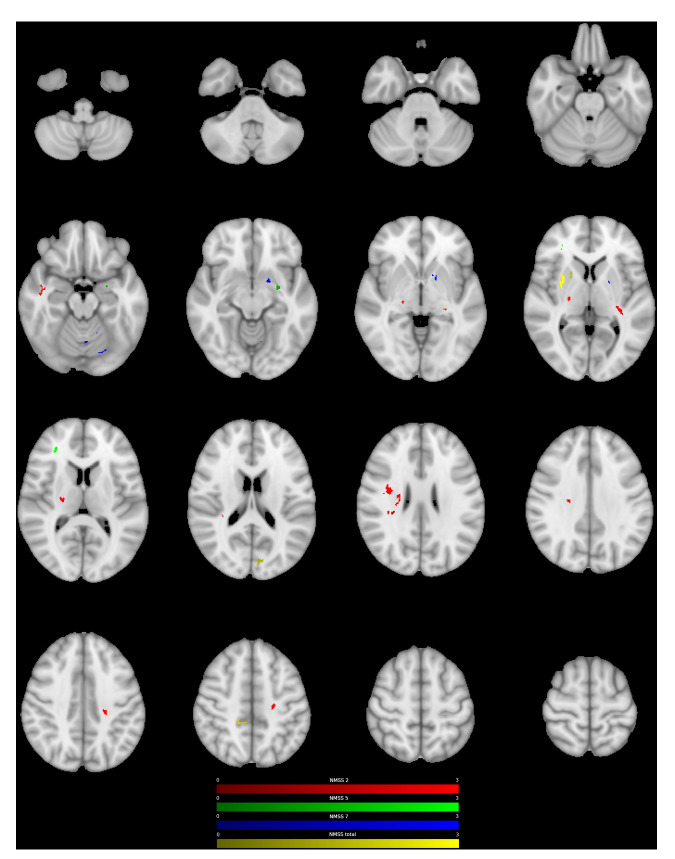
Positive association between ODI and postoperative change in NMS. Clusters with a positive association between PD patients’ ODI-values and postoperative change in NMSS-T (yellow), Domain 2 (sleep/fatigue, red), Domain 5 (attention/memory, green), and Domain 7 (urinary, blue), as revealed by the whole brain analysis. *P* values were corrected for multiple comparisons using a permutation-based approach.

**Fig. 2:**
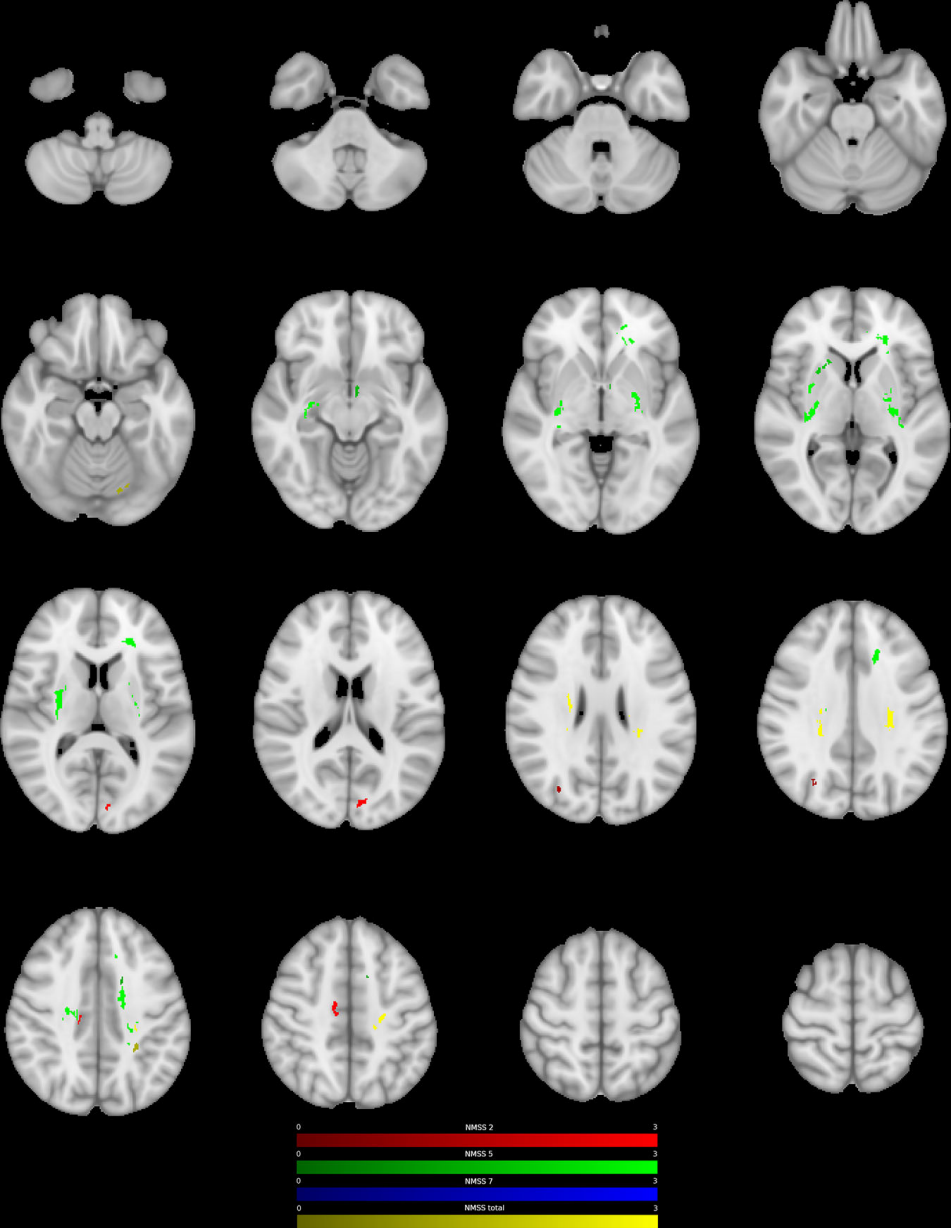
Negative association between ODI and postoperative change in NMS. Clusters with a negative association between PD patients’ ODI-values and postoperative change in NMSS-T (yellow), Domain 2 (sleep/fatigue, red), Domain 5 (attention/memory, green), and Domain 7 (urinary, blue), as revealed by the whole brain analysis. *P* values were corrected for multiple comparisons using a permutation-based approach.

We assessed the relationship between DTI metrics and postoperative non-motor symptom change using a generalised linear model corrected for multiple comparisons using a permutation-based approach. Higher fractional anisotropy (FA)-values in the right insular cortex were associated with greater postoperative NMSS-T reduction (positive cluster P1, cluster-wise *p* value (CWP): 0.032). Furthermore, lower FA-values were found in clusters including the bilateral cingulum and the left inferior longitudinal fasciculus (ILF), which were related to greater postoperative NMSS-T reduction (negative clusters N1-3, CWP: <0.001–0.014). Regional mean FA-values associated with the postoperative change in non-motor symptom burden are detailed in Supplementary Table 2 and Figs. 1 and 2.

### Interaction between NODDI-parameters and postoperative non-motor symptom change

Whole brain analysis of NODDI parameters showed that ODI and NDI were associated with changes in postoperative NMSS-T. Higher ODI-values in the right putamen, the right cingulate cortex, and the left forceps major were related to a higher postoperative NMSS-T reduction (P1-4, CWP: <0.001–0.034, Fig. 1, Supplementary Table 3). Furthermore, lower ODI-values in regions of both corticospinal tracts (CST) as well as left occipital fusiform gyrus were related to a higher postoperative NMSS-T reduction (N1-4, CWP: <0.001–0.047, Fig. 2, Supplementary Table 3). Lower NDI-values in the left postcentral gyrus, left cingulum, and right forceps minor were associated with a higher postoperative NMSS-T reduction (N1-4, CWP: <0.001–0.047, Supplementary Figure 4, Supplementary Table 4). No positive associations between NDI-values and postoperative NMSS-T change were detected.

### Interaction between microstructure and postoperative change in Non-Motor Symptoms Scale domains

Regional mean values of microstructural metrics associated with significant postoperative changes in non-motor symptoms scale domains are detailed in Supplementary Table 5 (sleep/fatigue), Supplementary Table 6 (attention/memory), and Supplementary Table 7 (urinary symptoms). Higher values of microstructural metrics within vast regions of bilateral CST were related to a higher symptom reduction in the sleep and fatigue domain (FA: P1-2, CWP: 0.001–0.005; ODI: P1-4, CWP: <0.001; NDI: P1, CWP: 0.004; Supplementary Table 5). With regard to attention and memory, higher FA-values in bilateral cingulum, left insular cortex, and left anterior thalamic radiation (AThR) were associated with higher postoperative symptom reduction (P1-4, CWP: <0.001–0.031, Supplementary Table 6). Furthermore, higher ODI-values in left parahippocampal gyrus and within the right frontal pole were related to positive postoperative outcomes in attention and memory (P1-2, CWP: <0.001–0.049), whereas lower ODI-values in bilateral AThR and left cingulum were associated with higher symptom reduction (N3, N5, N8-10, CWP: <0.001–0.039). Also, vast areas of bilateral CST and putamen that had lower ODI- and NDI-values were associated with higher symptom reduction in the attention/memory domain (ODI: N1, N2, N4 and N7-8, CWP: <0.001–0.011; NDI: N1-6 CWP: <0.001–0.023). Concerning urinary symptoms, higher ODI- and NDI-values in left putamen, pallidum, and AThR as well as left cerebellar lobule V and VI were related to higher postoperative symptom reduction in the urinary domain (ODI: P1-3, CWP: <0.001–0.031; NDI: P1-2, P4, CWP: <0.001–0.002, Supplementary Table 7). This association was also present between NDI-values and right pallidum and putamen (P3, CWP: 0.002). Furthermore, higher FA-values in the right cingulate gyrus and left superior longitudinal fasciculus (SLF) were associated with higher postoperative reduction of urinary symptoms (P1-2, CWP: <0.001–0.01).

### Interaction between microstructure and postoperative change in Apathy Evaluation Scale

Regional mean values of microstructural metrics associated with significant postoperative changes in apathy evaluation scale are detailed in Supplementary Table 8. Higher FA-values in right forceps major were related to a higher reduction of apathy (P1, CWP: <0.001). Furthermore, higher FA-values and lower ODI-values in right inferior fronto-occipital fasciculus and right inferior longitudinal fasciculus were associated with higher postoperative symptom reduction (FA: P2, CWP: .02; ODI: N1-2, N4, CWP: <0.001–0.006), whereby higher NDI-values in right parahippocampal gyrus were related to a higher reduction of apathy (P1, CWP: <0.001).

### Interaction between microstructure and postoperative non-motor symptom change controlled for the overlap between volume of tissue activated and STN

In a supplementary analysis, we reran the GLM-analysis and assessed the relationship between DTI metrics and postoperative non-motor symptom change while considering the volume of tissue activated (VTA) as a covariate. For conciseness, findings are displayed as supplementary figures 6 to 11. While certain smaller clusters did not survive correction for multiple comparisons when accounting for the VTA, we could replicate the main findings of our analysis for NMSS-T, as well as the non-motor symptoms scale domains.

## Discussion

In the present study, NODDI-DTI, a novel method for analyzing diffusion-weighted imaging (DWI) data, was applied to preoperative imaging to investigate cerebral microstructure associated with non-motor symptom changes following neurostimulation in PD. There are two key findings. First, we demonstrate that intact microstructure within specific areas, including right insular cortex, right putamen, right cingulum, and bilateral CST were associated with higher reduction of postoperative nonmotor symptom burden. Second, we delineate the structures and their microstructural properties, which are associated with postoperative improvements in specific non-motor domains.

Whole brain analysis of NODDI parameters identified an association between higher ODI in right putamen as well as right cingulum and a higher reduction of postoperative non-motor symptom burden. As ODI is high in grey matter^7^, this finding supports the notion that intact sprawling of dendritic processes in these areas is important for beneficial postoperative non-motor outcomes. In PD, the pathological deposition of alpha-synuclein in intraneuronal Lewy inclusions is accompanied by a degeneration of neurons and severe morphological changes of dendrites^13,14^. These tissue changes cannot be seen in conventional MRI, whereas NODDI is sensitive to neurite morphology^7^. Indeed, previous work demonstrated reduced putaminal ODI values in patients with PD compared to healthy controls^14^. This finding was interpreted as decreased dendrite length and loss of spines of striatal medium spiny neurons, which are the primary target of dopaminergic nigrostriatal projections^14^. Considering the close topographical relationship between STN and putamen as well as insular cortex and the integration of STN in basal ganglia-thalamo-cortical loops with its motor, associative, and limbic projections, it is important to examine the assumed mechanisms of action of DBS^5,9^. Besides effects on the micro- and mesoscale, the high-frequency pulses of electrical current emitted by DBS electrodes affect interregional networks on the macroscale^5^. Here, modulation of networks has been able to predict postoperative outcomes across several motor and non-motor symptoms^5^. Furthermore, previous work has shown that compromised putaminal microstructure in PD patients can be associated with higher non-motor symptom burden independent of motor symptoms^10^. Therefore, it can be hypothesised that the positive association between ODI and postoperative non-motor outcomes in the present study represents the dependency of DBS on intact tissue structure to exert its network effects.

Besides associations with grey matter areas, ODI showed a negative association with postoperative non-motor symptom burden in bilateral CST. In healthy white matter tissue, ODI is usually low, as fibres are highly coherent to another. High values of ODI, on the other hand, represent axonal disorganisation and degeneration^7^. Therefore, the observed negative association might reflect the dependence of DBS on intact tissue structure in CST, as low ODI, i.e. intact white matter microstructure, was associated with beneficial postoperative outcomes. Previous DTI-studies have repeatedly demonstrated microstructural alterations of CST in PD. In particular, increased FA has consistently been reported and hypothesised to demonstrate a compensatory mechanism, reflecting axonal sprouting secondary to a reduced input from striatum and thalamus^11^. When, however, microstructure in CST deteriorates over the course of the disease, an association with motor dysfunction was demonstrated^12^. Associations between altered CST-microstructure and non-motor symptoms, on the other hand, have received little attention. Employing a connectometry analysis in 85 patients, Ashraf-Ganjouei and colleagues could demonstrate that lower axonal density in CST was associated with a higher burden of gastrointestinal symptoms^15^. Furthermore, lower FA-values in right CST were observed in PD patients with depression compared to non-depressed patients^16^. Extending these findings, the results of the present study demonstrate that not only non-motor symptoms but also beneficial non-motor outcomes following STN-DBS depend on intact CST microstructure. Considering the role of the CST as major effector of motor control, motor outcomes and the postoperative reduction in dopaminergic medication could contribute to the beneficial non-motor effects in the present study. Importantly, however, postoperative improvements in non-motor symptom burden were not related to improvements in LEDD, LEDD-DA, and SCOPA-motor examination scores, which is in accordance with the literature^17,18^. Therefore, the association between intact CST microstructure and beneficial postoperative non-motor outcomes seems to be independent of the motor effects of DBS and suggests that CST microstructure is inherently relevant for non-motor symptoms in PD.

Sleep disturbances affect the majority of PD patients and result in poor quality of life^19^. Associated disorders encompass both, disturbances of sleep-wake transition, as well as parasomnias^19^. Although the exact neural mechanisms remain to be established, a disrupted interaction of neuronal circuits and different neurotransmitter systems has been suggested to underlie the sleep disturbances in PD^20,21^. STN-DBS has been suggested to improve sleep by alleviating motor symptoms and directly altering sleep physiology resulting in increased total sleep time, sleep efficiency, and quality of sleep as well as reduced wakefulness after sleep onset and insomnia^19^. In the present study, STN-DBS improved symptoms of the sleep/fatigue domain and beneficial outcomes were associated with higher FA-, ODI-, and NDI-values in vast regions of the CST. While there is generally a negative correlation between FA and ODI in white matter, specific instances may arise where elevated levels of both FA and ODI coexist. This is valid as different combinations of NDI and ODI can result in identical FA values^7^. Specifically, heightened FA values may occur when both NDI and ODI are high, whereby substantial adjustments in NDI are required to compensate for relatively minor changes in ODI to match the same FA value^7^. On the other hand, lower ODI- and NDI-values in these areas were associated with detrimental or below average response to STN-DBS. As most of the clusters were overlapping and within regions of high fibre crossing and dispersion, the selective degeneration of crossing fibres might underlie the observed relationship. The results, therefore, support the hypothesis that intact microstructure of the CST and its crossing fibres is important for beneficial postoperative changes in the sleep and fatigue domain. Additionally, high ODI-values of cortical structures including right superior and middle temporal gyrus and right parietal operculum cortex were associated with beneficial postoperative outcomes. Previous studies of healthy controls using simultaneous recordings of electroencephalography and functional MRI during sleep reported an association of activity increases within these areas with sleep spindles during early stages of non-rapid eye movement (NREM) sleep^22^. In PD, spindle density and amplitude seems to be reduced during NREM and modulated by dopaminergic therapy^19^. Integrating these findings with results of the present study one could speculate that intact microstructure, i.e. dendritic arborisation, in these areas is important for STN-DBS to modulate sleep physiology.

Higher FA (left hemisphere) and lower ODI in bilateral AThR were associated with beneficial postoperative outcomes in the attention and memory domain. These results suggest, that degenerative changes in axonal structure underlying FA alterations in AThR are attributable to changes in axonal fanning and dispersion. AThR connects the frontal lobe, the dorsolateral prefrontal cortex (DLPFC) in particular, with the anterior and midline nuclei of the thalamus^9^. As these nuclei are integrated in functional loops with the cingulum and the pallidum, the nuclei and their fibre connections are associated with the limbic system and thought to be involved in executive functions and planning of complex behaviour^9,23^. The DLPFC is involved in various higher-level cognitive functions, including attention, working memory, and executive control^24,25^. Considering the functional association of AThR with STN, as well as its role in connecting the structures named above, it seems reasonable that intact microstructure in AThR is important for the conveyance of beneficial effects on memory function and attention.

Higher FA and lower ODI in overlapping clusters in the bilateral cingulum as well as associated structures were related to increases in postoperative attention and memory function, indicating that axonal fanning and dispersion underlies alterations in FA in the cingulum. The cingulum is a group of nerve fibres connecting the hippocampus, prefrontal, parietal, and anterior cingulate cortex^26^. This allows for the integration of information from these structures and explains the involvement of the cingulum in attention, memory, and emotion regulation^26^. Previous studies in PD have shown that compromised microstructure of the cingulum was related to reduced scores in cognitive assessments, impaired visuospatial memory, and dementia^27^. Taken together, the association between microstructural properties and beneficial attention and memory outcomes in bilateral AThR and cingulum might represent the dependency of DBS on intact tissue structure to exert its network effects.

Deficient perception of multimodal sensory information is a characteristic of PD leading to debilitating non-motor symptoms^28^. Sensory deficiencies in PD have been described in both, somatosensory pathways associated with proprioception as well as visceral pathways involved in monitoring of urinary bladder filling^29^. STN-DBS in PD was shown to improve the perception of urinary bladder filling, resulting in a delayed desire to void and increased bladder capacity^28^. Improved urinary function was attributed to a beneficial influence of STN-DBS on a gain of afferent bladder information due to an increase or decrease of activation of primary sensory areas^28^. In particular, previous studies showed an activation of anterior cingulate gyrus (ACC) and left lateral frontal cortex during monitoring and controlling the storage phase of the urinary cycle and these structures are thought to be involved in the urge to void, withholding urine, and the onset of micturition^28^. In the present study, intact microstructure, i.e. high FA, in right ACC was associated with postoperative improvements in urinary symptoms suggesting that sound microstructure in ACC is necessary for STN-DBS to have beneficial effects on controlling the storage phase of the urinary cycle. Furthermore, high NDI and ODI, i.e. high dispersion without a decrease in axonal density, in left AThR was associated with positive outcomes of urinary symptoms. AThR connects the frontal cortex with the thalamus and the pallidum and previous studies suggested that modulation of left frontal cortex by STN-DBS is important for urge control^28^. Therefore, intact microstructure in left AThR might be relevant for STN-DBS to modulate left frontal cortex during urge control^28^. Further important structures implicated in processing afferent urinary bladder information are the posterior thalamus, which is activated during bladder filling and micturition, and the ventrolateral, as well as reticular thalamus, which receive input from the striatum to modulate the flow of visceral information between the posterior thalamus and the cortex. Previous studies speculated that STN-DBS may recondition the interaction between pallidal output and the modulatory effect of the thalamus, resulting in improved gating of sensory information^28^. One might speculate, that the association of intact microstructure in left pallidum and putamen with better outcomes in the urinary domain represents the importance of these structures for the effect of STN-DBS on gating sensory information.

Apathy is characterised by deficiencies in the initiation, enactment, and achievement of goal-directed behaviour and represents a debilitating neuropsychiatric condition frequent in advanced stages of PD^30,31^. The disruption of networks connecting the frontal cortex with limbic structures, along with alterations in callosal interhemispheric connections and long-range non-dopaminergic projections, have been linked with apathy in PD^30^. Following STN-DBS, apathy is a frequent symptom in PD patients, whereby recent findings indicate that neither reduction of dopaminergic therapy nor subthalamic stimulation are associated with postoperative apathy^32^. In the present study, intact microstructure in the right forceps major and long-range fibre bundles connecting the frontal and parieto-occipital lobes were associated with beneficial outcomes in apathy. Furthermore, preserved axonal organisation, i.e. high NDI, in the anterior division of the right parahippocampal gyrus was linked to postoperative improvements in apathy scores. These findings suggest that the beneficial effects of STN-DBS on apathy depend on the integrity of the microstructure of interhemispheric and long-range fronto-parieto-occipital connections as well as structures within the right limbic system.

This research has its limitations. First, despite histopathological validation of the NODDI model and its frequent use in PD, no studies validating the model in post-mortem brain tissue of PD patients exist. Second, the underlying assumptions of the NODDI model may represent an oversimplification and might therefore result in reduced specificity^7^. It has been shown, however, that the underlying biophysiological model supports a realistic description of tissue structure at a clinically feasible acquisition time^7,33^. Third, motor phenotype such as tremor-dominant or postural instability and gait difficulty (PIGD) can have an impact on non-motor outcomes^34^. The sample size of the present study, however, impeded a more detailed delineation of the outcomes based on motor phenotype. Fourth, the resolution of the DTI scan in our study is limited to 2.0 × 2.0 × 2.0 mm³. This resolution, however, is similar to previous NODDI studies^35–37^ and was chosen to find a compromise between scanning time, image resolution, and signal-to-noise ratio, where longer durations in the scanner lead to more noticeable motion artefacts, a crucial consideration in PD. Fifth, we refrained from utilizing scales designed exclusively for assessing distinct non-motor symptoms, such as the Parkinson’s Disease Sleep Scale (PDSS) for sleep-related issues. This decision was driven by our interest in investigating the association between microstructure and the change in overall non-motor symptom burden. Furthermore, the NMSS is the most established scale of NMS in PD, comprehensively covering different NMS related to the key neurotransmitters involved in the pathogenesis of PD^20^. It has been employed as both a primary and secondary endpoint in a multitude of clinical trials, finds extensive utilization within clinical settings, and has demonstrated strong test-retest reliability^20,38^. These qualities collectively establish it as a pertinent instrument for investigating alterations in non-motor symptoms, facilitating comparability across different studies, and serving a practical role in clinical applications. Furthermore, the general limitations of open-label studies are inherent to this study as well. Despite several advantages of this study design, open-label studies have several limitations including placebo effects that can significantly influence subjective measures and contribute to overall patient well-being. Furthermore, the inclusion of a control group is not practicable in open-label DBS studies. Nevertheless, the use of clinician rated forms, as employed in this study, can help to overcome the challenges named above and to minimize bias.

In conclusion, we describe a spatially distinct profile of microstructural alterations associated with beneficial non-motor outcomes following neurostimulation in PD, including right insular cortex, putamen, cingulum, and bilateral CST. Furthermore, we delineate the structures and their microstructural properties, which are associated with postoperative improvements in specific non-motor domains. These findings remain consistent even after accounting for post-operative factors such as the overlap between the volume of tissue activated and the STN. Therefore, we suggest that diffusion MRI can support preoperative patient counselling by identifying patients with above- or below-average non-motor responses.

## Methods

The study was approved by the local ethics committee (study-number: 155/17) and carried out following the Declaration of Helsinki.

### Participants

Thirty-seven PD patients (9 female, mean age 58.8 ± 7.3 years) were enrolled in this prospective, observational, ongoing study upon written informed consent (for demographics cf. Table 1). Inclusion criteria comprised indication for DBS lead surgery because of advanced PD according to modern criteria^39^. Patients were excluded if they had pathological MRI, a concomitant neurological or psychiatric disease, or impaired visual or auditory function.

### Clinical assessment

Patients were assessed preoperatively and six months after DBS surgery, with medication at both times and DBS switched on postoperatively. Demographic and clinical data were collected on both study visits for each participant using standardized case report forms. Patients underwent a comprehensive neuropsychological assessment including the following scales:

Non-motor Symptoms Scale (NMSS): the clinician-rated scale comprises 30 items evaluating 9 dimensions of non-motor symptoms including (1) cardiovascular, (2) sleep/fatigue, (3) mood/cognition, (4) perceptual problems/hallucinations, (5) attention/memory, (6) gastrointestinal tract, (7) urinary, (8) sexual function, and (9) miscellaneous which in turn asks about pain, the ability to smell/taste, weight change, and sweating. The score ranges from 0 (no impairment) to 360 (maximum impairment) and assesses the NMS over the past 4 weeks^38^.

PD Questionnaire (PDQ)-8: the questionnaire is a short form of the PDQ-39 and determines eight dimensions of quality of life (QoL) in PD patients^40^. The score is a well-established measure in PD patients undergoing DBS surgery and reported as a summery index (SI) with a score range from 0 (no impairment) to 100 (maximum impairment)^1,2,18^.

Scales for Outcomes in PD (SCOPA) – Motor Function: the clinician-rated scale evaluates 3 dimensions of motor function in PD, comprising (A) motor evaluation, (B) activities of daily living, and (C) motor complications, with subscale scores ranging from 0 (no impairment) to 42 (motor evaluation), 21 (activities of daily living), and 12 (motor complications), respectively^41^.

Apathy Evaluation Scale (AES): the self-rated scale comprises 18 items quantifying and characterizing apathy^42^. The score is employed in adults with various neurocognitive disorders, including PD, and ranges from 18 to 72, with higher scores indicating more apathy.

Levodopa equivalent daily dose was calculated according to Tomlinson et al.^43^.

### MRI Data Acquisition and Processing

PD patients were scanned at baseline on a 3-Tesla Trio scanner (Siemens, Erlangen, Germany) at the Core Unit Brain Imaging of the Philipps-University Marburg. The acquisition protocol comprised the following sequences:

1. 3D T1-weighted Magnetization Prepared - RApid Gradient Echo sequence (MPRAGE, field of view (FoV) = 256 mm, matrix 256 × 256, 176 slices, slice thickness 1 mm, voxel dimension 1.0 × 1.0 × 1.0 mm³, repetition time (TR) = 1900 ms, echo time (TE) = 2.26 ms, inversion time (TI) = 900 ms, flip-angle = 9°, bandwidth (BW) = 200 Hz/Pixel, parallel imaging (GRAPPA) with factor 2)
2. diffusion-weighted imaging (DWI) (FoV = 256 mm, matrix 128 × 128, slice thickness 2 mm, distance factor 0%, voxel dimension 2.0 × 2.0 × 2.0 mm³, TR = 7900 ms, TE = 90 ms, BW = 1502 Hz/Pixel, 42 diffusion encoding gradients, three intermittent non-weighted b0 images (*b* = 0 s/mm²), high *b* value *b* = 1000 s/mm², GRAPPA with factor 2).

All images were investigated to be free of subject motion or ghosting and high frequency and/or wrap-around artefacts at the time of image acquisition.

### Image Processing

Image analysis was performed within the FreeSurfer image analysis suite 7.1.1 (http://surfer.nmr.mgh.harvard.edu) as reported previously by our group^44^. The processing of T1-weighted scans included skull stripping, automated Talairach transformation, cortical and subcortical segmentation, intensity normalisation, tessellation of the grey/white matter boundary, automated topology correction, and surface deformation following intensity gradients^45^.

DTI data were processed using FMRIB Software Library (FSL) 6.0.5.2 (https://fsl.fmrib.ox.ac.uk/fsl). To correct for eddy-current distortions and involuntary movements, raw DTI volumes were linearly registered and resampled to the first b0 volume^46^. Subsequently, the diffusion tensor for each voxel was fit to the data using linear regression and FA was derived from the diffusion tensor^47^. Furthermore, NODDI-DTI^48,49^, a modification of NODDI^7^, was used to obtain NDI and ODI from the DTI data. Visual inspection of the b0-images confirmed that no changes beyond those in the tissue structure contributed to the observed effects. To perform regional analyses, the first b0 image of each scan was linearly registered to the structural T1-weighted image using a boundary-based method, yielding an affine matrix^50^. Subsequently, T1-derived segmentations and brain masks were transformed to the diffusion space via the inverse of the affine matrix.

To confirm that electrodes were placed correctly and to control for the effect of volume of tissue activated on non-motor outcomes, we conducted a supplementary analysis and reconstructed the electrodes employing the Lead-DBS toolbox with default parameters (www.lead-dbs.org). In brief, advanced normalization tools (ANTs, http://stnava.github.io/ANTs/) was used to linearly coregister postoperative CT images to preoperative MRI. Subsequently, images were nonlinearly normalized into standard space (ICBM 2009b NLIN, Asym) and the PaCER algorithm was used to reconstruct electrodes (Supplementary Figure 5). Then, individual VTAs were modeled and the average percentage overlap between the VTA and the bilateral STN was calculated (Supplementary Table 9).

### Statistical analysis

Statistical analysis of clinical outcomes was performed in MATLAB (The MathWorks, Inc., R2018a). Changes between baseline and follow-up were analysed using the Wilcoxon signed-rank or t-test if parametric test criteria were met. The type-I error was controlled using the Benjamini-Hochberg method^51^ and effect sizes were calculated according to Cohen^52^ for parametric and Rosenthal^53^ for non-parametric tests. Relationships between change scores of clinical data and NMSS-T were explored using Spearman correlations as reported previously^34,54^.

Statistical voxelwise analysis of image data was performed using a generalized linear model. First, FA-maps were co-registered to the Montreal Neurological Institute and Hospital data space (MNI152) using linear and nonlinear transformation^55^. Masked FA-, NDI-, and ODI-maps were subsequently registered to the MNI152 space using the transformation from the previous step. Only voxels of brain tissue existing in every subject were included in the analysis. Significant associations between metrics of microstructure and change of non-motor symptoms were carried out for the whole brain as described previously^56^. Here, percentage differences between baseline and follow-up values were calculated for NMSS-T, NMSS domains with significant postoperative improvement ((2) sleep/fatigue, (5) attention/memory, and (7) urinary), and, in a complementary analysis, apathy evaluation scale. A permutation-based approach based on the Analysis of Functional NeuroImages (AFNI) null-z simulator was used to corrected for multiple comparisons employing 12,000 simulations under the null hypothesis^57^. Clusters were formed using a threshold of *p* < 0.01 and a clusterwise *p* value was calculated. Results were accepted as significant with clusterwise *p* < 0.05.

In a supplementary analysis, we reran the statistical voxelwise analysis incorporating the average percentage overlap between the VTA and the bilateral STN as a covariate (c.f. supplementary material).

### Reporting summary

Further information on research design is available in the Nature Research Reporting Summary linked to this article.

### Supplementary information

Supplementary Material

Reporting Summary

## Data Availability

The data supporting this study’s findings are available on request from the corresponding author (PAL). The data are not publicly available due to privacy or ethical restrictions.

## Code availability

All tools used to analyse MRI data are based on FreeSurfer Version 7.1 (http://surfer.nmr.mgh.harvard.edu/) and FSL 6.0.5.2 (http://www.fmrib.ox.ac.uk/fsl) packages, which are freely available. Scripts for automation were written in tcshell and parts of the statistics were written in Python using the packages numpy, pandas, seaborn, matplotlib, nibabel and scipy, which are also freely available. Python program code for the analysis of NODDI-DTI is available from https://github.com/dicemt/DTI-NODDI.
