# Supplementary material for: Microstructure predicts non-motor outcomes following deep brain stimulation in Parkinson’s disease
Source: NPJ Parkinsons Dis. 2024 May 18;10:104. doi: 10.1038/s41531-024-00717-y (PMC11102428; doi:10.1038/s41531-024-00717-y)
Supplement: Supplementary file 1 — Supplementary Material [file 41531_2024_717_MOESM1_ESM.pdf]

Supplementary Material

Supplementary Results

Correlation analysis

Supplementary Table 1 Correlations between change scores of clinical data and NMSS-T

	NMSS-Total change score		
Change scores	N	*p*-value	*effect size*
PDQ-8 Summary Index	37	.117	.26
SCOPA-			
Total	37	**.037**	**.34**
Motor evaluation	37	.288	.18
Activities of daily living	37	**.009**	**.43**
Motor complications	37	.495	.16
LEDD total	37	.417	.14
LEDD-DA	37	.72	-.06

Spearman correlations between change scores (baseline - 6-month follow-up) of clinical data and NMSS-T. Bold font highlights significant results, $p<.05$. Positive correlations indicate that higher changes in clinical scores are associated with higher changes in NMSS-T.

Abbreviations: LEDD: Levodopa Equivalent Daily Dose; LEDD-DA: LEDD of Dopamine Agonists; NMSS-T: Non-Motor Symptom Scale total score; PDQ-8 SI: = 8-item Parkinson's Disease Questionnaire summary index; SCOPA = Scales for Outcome in Parkinson's Disease.

Image analysis

Supplementary Table 2 Association between FA-Values and NMSS-T

Positive Cluster	Location	Slope	Intercept	p-Value	Volume in mm³	MNI152-Coordinates		
						X	Y	Z
P1	Right insular cortex	.0002	.1336	.032	183	40	-17	7
Negative Cluster								
N1	Right cingulum	-.0004	.4038	< .001	436	13	28	31
N2	Left Heschl's gyrus Left inferior longitudinal fasciculus	-.0006	.3364	< .001	282	-40	-26	5
N3	Left cingulum	-.0004	.4528	.014	203	-13	28	18

Supplementary Table 2. Characteristics of clusters with an association between PD patients' FA-values and postoperative change in NMSS-T. "Positive Cluster" denotes clusters with a positive association between patients' FA-values and percentage differences in NMSS-T, i.e. higher FA-values were associated with higher postoperative values. "Negative Cluster" denotes clusters with a negative association between patients' FA-values and percentage difference of NMSS-T, i.e. higher FA-values were associated with lower postoperative values. "Location" indicates the anatomical landmark comprising the majority of voxels of a cluster according to Johns Hopkins University (JHU) white matter atlas, Harvard-Oxford cortical and subcortical atlas, and University College London (UCL) cerebellar atlas. P-Values are clusterwise p-values corrected for multiple comparisons. "Volume in mm³" denotes the size of a cluster and "MNI152-coordinates" describes the coordinates of the cluster's center of gravity in MNI152-space.

Supplementary Table 3 Association between ODI-Values and NMSS-T

Positive Cluster	Location	Slope	Intercept	p-Value	Volume in mm³	MNI152-Coordinates		
						X	Y	Z
P1	Right putamen<br>Right superior longitudinal fasciculus	.0003	.3574	< .001	223	31	-6	7
P2	Right precuneous cortex<br>Right cingulate gyrus (posterior division)	.0008	.2567	.008	159	6	-36	43
P3	Right putamen<br>Right anterior thalamic radiation	.0004	.3853	.024	141	19	13	-2
P4	Left forceps major<br>Left cuneal cortex<br>Left intracalcarine cortex	.0003	.2911	.034	135	-11	-79	18
Negative Cluster								
N1	Left corticospinal tract	-.0002	.1987	< .001	785	-25	-17	29
N2	Right corticospinal tract	-.0002	.2191	< .001	306	23	-10	25
N3	Left superior longitudinal fasciculus<br>Left superior parietal lobule	-.0002	.2007	.038	134	-28	-40	37
N4	Left occipital fusiform gyrus	-.0003	.3549	.047	130	-15	-74	-15

Supplementary Table 3. Characteristics of clusters with an association between PD patients' ODI-values and postoperative change in NMSS-T. "Positive Cluster" denotes clusters with a positive association between patients' ODI-values and percentage differences in NMSS-T, i.e.

higher ODI-values were associated with higher postoperative values. “Negative Cluster” denotes clusters with a negative association between patients’ ODI-values and percentage difference of NMSS-T, i.e. higher ODI-values were associated with lower postoperative values. “Location” indicates the anatomical landmark comprising the majority of voxels of a cluster according to Johns Hopkins University (JHU) white matter atlas, Harvard-Oxford cortical and subcortical atlas, and University College London (UCL) cerebellar atlas. P-Values are clusterwise p-values corrected for multiple comparisons. “Volume in mm³” denotes the size of a cluster and “MNI152-coordinates” describes the coordinates of the cluster’s center of gravity in MNI152-space.

Supplementary Table 4 Association between NDI-Values and NMSS-T

Negative Cluster	Location	Slope	Intercept	p-Value	Volume in mm³	MNI152-Coordinates		
						X	Y	Z
N1	Left postcentral gyrus<br>Left superior longitudinal fasciculus	-.0004	.5663	< .001	273	-33	-31	47
N2	Left cingulum	-.0003	.5228	.009	157	-7	-25	20
N3	Right forceps minor	-.0006	.4678	.049	125	12	19	15

Supplementary Table 4. Characteristics of clusters with an association between PD patients’ NDI-values and postoperative change in NMSS-T. “Negative Cluster” denotes clusters with a negative association between patients’ NDI-values and percentage difference of NMSS-T, i.e. higher NDI-values were associated with lower postoperative values. “Location” indicates the anatomical landmark comprising the majority of voxels of a cluster according to Johns Hopkins University (JHU) white matter atlas, Harvard-Oxford cortical and subcortical atlas, and University College London (UCL) cerebellar atlas. P-Values are clusterwise p-values corrected

for multiple comparisons. "Volume in mm³" denotes the size of a cluster and "MNI152-coordinates" describes the coordinates of the cluster's center of gravity in MNI152-space.

Domain specific Statistics

Supplementary Table 5 Association between microstructural metrics and sleep/fatigue-outcomes

**Fractional Anisotropy**								
Positive Cluster	Location	Slope	Intercept	p-Value	Volume in mm³	MNI152-Coordinates		
						X	Y	Z
P1	Right superior longitudinal fasciculus	.0003	.3536	.001	269	27	-40	32
P2	Right postcentral gyrus	.0002	.1475	.005	233	55	-19	21
Negative Cluster								
N1	Left inferior longitudinal fasciculus<br>Left temporal fusiform cortex	-.0003	.2373	.02	194	-41	-38	-12
N2	Right parietal operculum cortex	-.0002	.3547	.031	184	32	-31	20
**Orientation Dispersion Index**								
Positive Cluster								
P1	Right corticospinal tract	.0001	.0858	< .001	297	24	-17	8
P2	Right superior longitudinal fasciculus<br>Right corticospinal tract	.0002	.2448	< .001	297	35	-5	25

P3	Left corticospinal tract	.0001	.1810	< .001	270	-26	-22	40
P4	Right corticospinal tract	.0001	.1694	< .001	224	24	-19	29
P5	Right planum polare<br>Right middle temporal gyrus	.0002	.2597	.002	181	46	-12	-16
P6	Left inferior fronto-occipital fasciculus<br>Left inferior longitudinal fasciculus	.0001	.1169	.002	179	-30	-27	3
P7	Right parietal operculum cortex	.0001	.2207	.009	157	37	-33	21
Negative Cluster								
N1	Right precentral gyrus	-.0004	.4102	< .001	223	11	-13	45
N2	Left cuneal cortex<br>Left forceps major	-.0004	.3312	.001	189	-11	-79	18
N3	Right lateral occipital cortex	-.0004	.2939	.043	131	30	-71	26
**Neurite Density Index**								
Positive Cluster								
P1	Right corticospinal tract	.0002	.5713	.004	166	26	-20	25

Supplementary Table 5. Characteristics of clusters with an association between PD patients' microstructural metrics and postoperative change in NMSS-Domain 2 (Sleep/Fatigue). "Positive Cluster" denotes clusters with a positive association between patients' microstructural metrics and postoperative changes in NMSS-Domain 2, i.e. higher values of a specific metric were associated with higher postoperative values. "Negative Cluster" denotes clusters with a negative association between patients' microstructural metrics and postoperative difference in NMSS-Domain 2, i.e. higher values of a specific metric were associated with lower

postoperative values. “Location” indicates the anatomical landmark comprising the majority of voxels of a cluster according to Johns Hopkins University (JHU) white matter atlas, Harvard-Oxford cortical and subcortical atlas, and University College London (UCL) cerebellar atlas. P-Values are clusterwise p-values corrected for multiple comparisons. “Volume in mm³” denotes the size of a cluster and “MNI152-coordinates” describes the coordinates of the cluster’s center of gravity in MNI152-space.

Supplementary Table 6 Association between microstructural metrics and attention/memory-outcomes

**Fractional Anisotropy**								
Positive Cluster	Location	Slope	Intercept	p-Value	Volume in mm³	MNI152-Coordinates		
						X	Y	Z
P1	Left anterior thalamic radiation	.0002	.3534	< .001	319	-26	26	5
P2	Right precentral gyrus<br>Right cingulate gyrus	.00017	.1418	.01	211	13	-18	37
P3	Left insular cortex<br>Left inferior fronto-occipital fasciculus	.00018	.45	.012	205	-30	-22	-2
P4	Left cingulum	.00018	.3651	.031	185	-17	24	27
Negative Cluster								
N1	Right superior longitudinal fasciculus	-.00021	.4261	.006	225	33	7	20
**Orientation Dispersion Index**								
Positive Cluster								
P1	Right frontal pole	.0002	.2707	< .001	203	30	37	6
P2	Left parahippocampal gyrus	.0002	.1944	.049	128	-27	-7	-10

	Left inferior fronto-occipital fasciculus							
Negative Cluster								
N1	Right Putamen Right cerebral white matter	-.0002	.2699	< .001	1782	35	-24	2
N2	Left Putamen Left cerebral white matter	-.0003	.2902	< .001	916	-29	-18	-1
N3	Left anterior thalamic radiation	-.0002	.2264	< .001	734	-23	32	7
N4	Right corticospinal tract	-.0001	.2051	< .001	368	18	-16	31
N5	Left cingulum	-.0002	.2440	< .001	267	-16	23	30
N6	Left cerebral white matter	-.0002	.2181	< .001	248	-16	-5	37
N7	Left corticospinal tract	-.0001	.1990	.002	187	-28	-23	38
N8	Right anterior thalamic radiation Right Putamen	-.0002	.4027	.011	153	26	9	3
N9	Left anterior thalamic radiation Left cerebral white matter	-.0002	.2531	.023	141	-5	-3	-9
N10	Left cerebral white matter	-.0002	.2233	.039	133	-16	13	37
**Neurite Density Index**								
Negative Cluster								
N1	Right Putamen	-.0004	.6042	< .001	1380	31	-18	6
N2	Right precentral gyrus Right superior	-.0003	.6311	< .001	450	28	-12	41

	longitudinal fasciculus							
N3	Left Putamen	-.0006	.6524	< .001	234	-23	-8	7
N4	Right corticospinal tract	-.0002	.6063	< .001	232	25	-14	16
N5	Right corticospinal tract	-.0003	.6590	.009	152	15	-32	-26
N6	Left corticospinal tract	-.0004	.6523	.023	138	1	-31	-22
N7	Right V	-.0003	.6293	.031	133	6	-61	-22

Supplementary Table 6. Characteristics of clusters with an association between PD patients' microstructural metrics and postoperative change in NMSS-Domain 5 (Attention/Memory). "Positive Cluster" denotes clusters with a positive association between patients' microstructural metrics and postoperative changes in NMSS-Domain 5, i.e. higher values of a specific metric were associated with higher postoperative values. "Negative Cluster" denotes clusters with a negative association between patients' microstructural metrics and postoperative difference in NMSS-Domain 5, i.e. higher values of a specific metric were associated with lower postoperative values. "Location" indicates the anatomical landmark comprising the majority of voxels of a cluster according to Johns Hopkins University (JHU) white matter atlas, Harvard-Oxford cortical and subcortical atlas, and University College London (UCL) cerebellar atlas. P-Values are clusterwise p-values corrected for multiple comparisons. "Volume in mm^3" denotes the size of a cluster and "MNI152-coordinates" describes the coordinates of the cluster's center of gravity in MNI152-space.

Supplementary Table 7 Association between microstructural metrics and urinary-outcomes

**Fractional Anisotropy**								
Positive Cluster	Location	Slope	Intercept	p-Value	Volume in mm³	MNI152-Coordinates		
						X	Y	Z
P1	Right cingulate gyrus, anterior division	.0003	.3113	< .001	273	8	19	26
P2	Left superior longitudinal fasciculus	.0003	.1206	.01	209	-43	-37	22
Negative Cluster								
N1	Left inferior fronto-occipital fasciculus	-.0004	.2375	.004	235	-32	32	3
**Orientation Dispersion Index**								
Positive Cluster								
P1	Left Pallidum<br>Left Putamen<br>Left anterior thalamic radiation	.0003	.2993	< .001	198	-16	2	-6
P2	Left VI	.0003	.3427	.005	167	-17	-70	-18
P3	Left V	.0004	.2985	.031	137	-11	-60	-10
**Neurite Density Index**								
Positive Cluster								
P1	Left Pallidum<br>Left Putamen<br>Left anterior thalamic radiation	.0006	.5718	< .001	226	-15	2	-7
P2	Left VI	.0005	.4453	< .001	193	-13	-75	-15
P3	Right Pallidum<br>Right Putamen	.0003	.5266	.002	177	12	10	-10

P4	Left V	.0006	.4012	.002	172	-5	-62	-9

Supplementary Table 7. Characteristics of clusters with an association between PD patients' microstructural metrics and postoperative change in NMSS-Domain 7 (Urinary). "Positive Cluster" denotes clusters with a positive association between patients' microstructural metrics and postoperative changes in NMSS-Domain 7, i.e. higher values of a specific metric were associated with higher postoperative values. "Negative Cluster" denotes clusters with a negative association between patients' microstructural metrics and postoperative difference in NMSS-Domain 7, i.e. higher values of a specific metric were associated with lower postoperative values. "Location" indicates the anatomical landmark comprising the majority of voxels of a cluster according to Johns Hopkins University (JHU) white matter atlas, Harvard-Oxford cortical and subcortical atlas, and University College London (UCL) cerebellar atlas. P-Values are clusterwise p-values corrected for multiple comparisons. "Volume in mm^3" denotes the size of a cluster and "MNI152-coordinates" describes the coordinates of the cluster's center of gravity in MNI152-space.

Apathy evaluation scale Statistics

Supplementary Table 8 Association between microstructural metrics and apathy-outcomes

**Fractional Anisotropy**								
Positive Cluster	Location	Slope	Intercept	p-Value	Volume in mm³	MNI152-Coordinates		
						X	Y	Z
P1	Right forceps major	.001	.678	< .001	479	24	-54	12
P2	Right lateral occipital cortex, Right inferior fronto-occipital fasciculus	.0009	.4103	.02	200	21	-57	38
Negative Cluster								
N1	Left parahippocampal gyrus, anterior division	-.0006	.1554	< .001	355	-21	-25	-24
**Orientation Dispersion Index**								
Negative Cluster								
N1	Right inferior fronto-occipital fasciculus	-.0005	.0966	< .001	236	32	-65	20
N2	Right inferior fronto-occipital fasciculus	-.0006	.1821	< .001	227	20	-54	37
N3	Right anterior thalamic radiation	-.0005	.1403	.004	175	30	-24	-4
N4	Right inferior longitudinal fasciculus	-.0008	.1789	.006	165	45	-40	-10
**Neurite Density Index**								
Positive Cluster								
P1	Right parahippocampal gyrus, anterior division	.0014	.4543	< .001	202	32	-31	-11

Supplementary Table 8. Characteristics of clusters with an association between PD patients' microstructural metrics and postoperative change in apathy evaluation scale (AES). "Positive Cluster" denotes clusters with a positive association between patients' microstructural metrics and postoperative changes in AES, i.e. higher values of a specific metric were associated with higher postoperative values. "Negative Cluster" denotes clusters with a negative association between patients' microstructural metrics and postoperative difference in AES, i.e. higher values of a specific metric were associated with lower postoperative values. "Location" indicates the anatomical landmark comprising the majority of voxels of a cluster according to Johns Hopkins University (JHU) white matter atlas, Harvard-Oxford cortical and subcortical atlas, and University College London (UCL) cerebellar atlas. P-Values are clusterwise p-values corrected for multiple comparisons. "Volume in mm^3" denotes the size of a cluster and "MNI152-coordinates" describes the coordinates of the cluster's center of gravity in MNI152-space.

Supplementary Table 9 Overlap between VTA and bilateral STN

Patient 1	19
Patient 2	53
Patient 3	70
Patient 4	57
Patient 5	60
Patient 6	80
Patient 7	34
Patient 8	88
Patient 9	32
Patient 10	13
Patient 11	35
Patient 12	54
Patient 13	75
Patient 14	82
Patient 15	33
Patient 16	82
Patient 17	70
Patient 18	27
Patient 19	71
Patient 20	2
Patient 21	62
Patient 22	42
Patient 23	69
Patient 24	20
Patient 25	99
Patient 26	44
Patient 27	12
Patient 28	65
Patient 29	42
Patient 30	18
Patient 31	30
Patient 32	24
Patient 33	50
Patient 34	80
Patient 35	2
Patient 36	50
Patient 37	42

Supplementary Table 9. Average percentage overlap between the VTA and bilateral STN (rounded to no decimal place).

Supplementary Figures

Supplementary Figure 1.

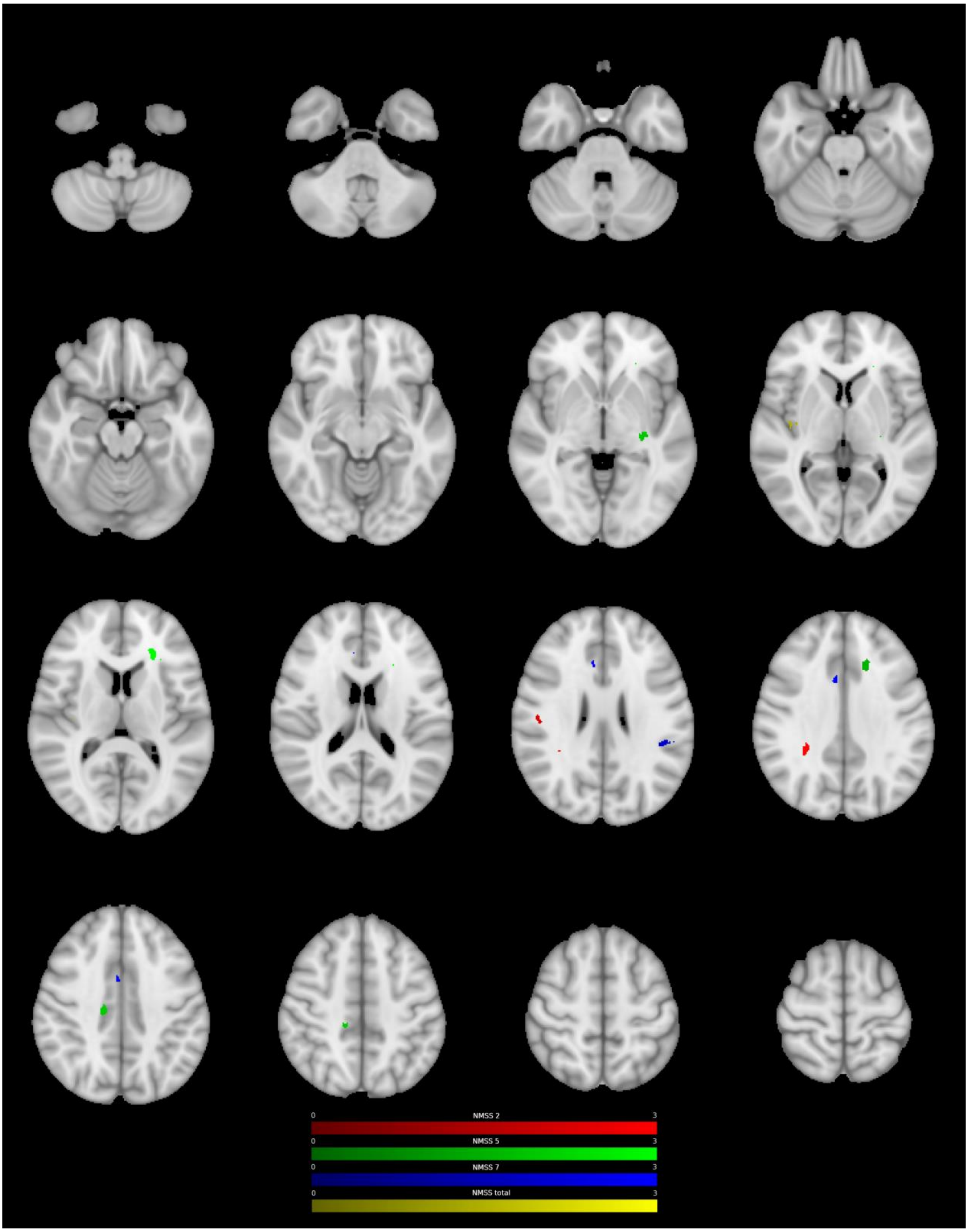

Supplementary Figure 1. Clusters with a positive association between PD patients' FA-values and postoperative change in NMSS-T (yellow), Domain 2 (sleep/fatigue, red), Domain 5 (attention/memory, green), and Domain 7 (urinary, blue), as revealed by the whole brain

analysis. P-Values were corrected for multiple comparisons using a permutation-based approach.

Supplementary Figure 2

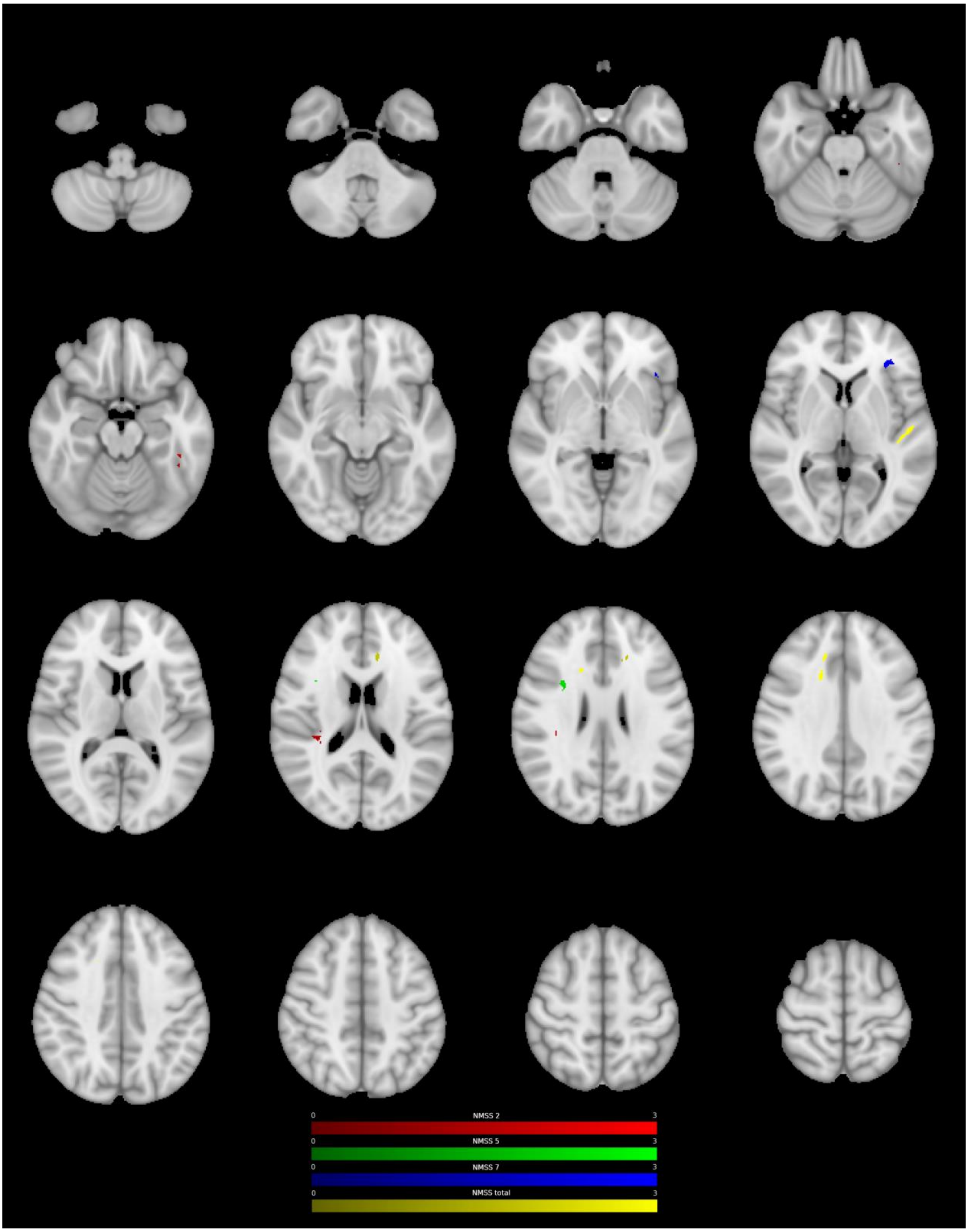

Supplementary Figure 2. Clusters with a negative association between PD patients' FA-values and postoperative change in NMSS-T (yellow), Domain 2 (sleep/fatigue, red), Domain 5

(attention/memory, green), and Domain 7 (urinary, blue), as revealed by the whole brain analysis. P-Values were corrected for multiple comparisons using a permutation-based approach.

Supplementary Figure 3

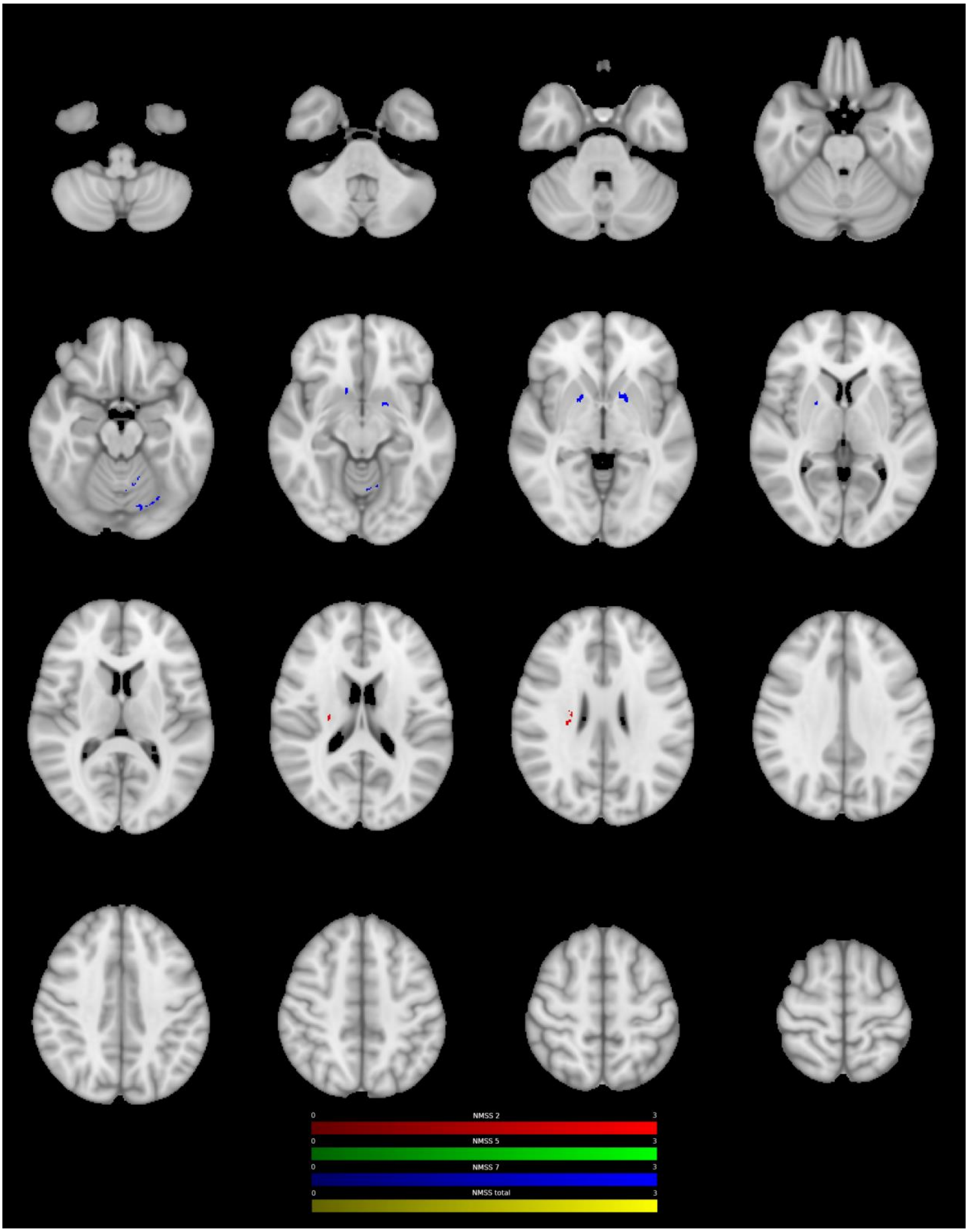

Supplementary Figure 3. Clusters with a positive association between PD patients' NDI-values and postoperative change in NMSS-T (yellow), Domain 2 (sleep/fatigue, red), Domain 5 (attention/memory, green), and Domain 7 (urinary, blue), as revealed by the whole brain analysis. P-Values were corrected for multiple comparisons using a permutation-based approach.

Supplementary Figure 4

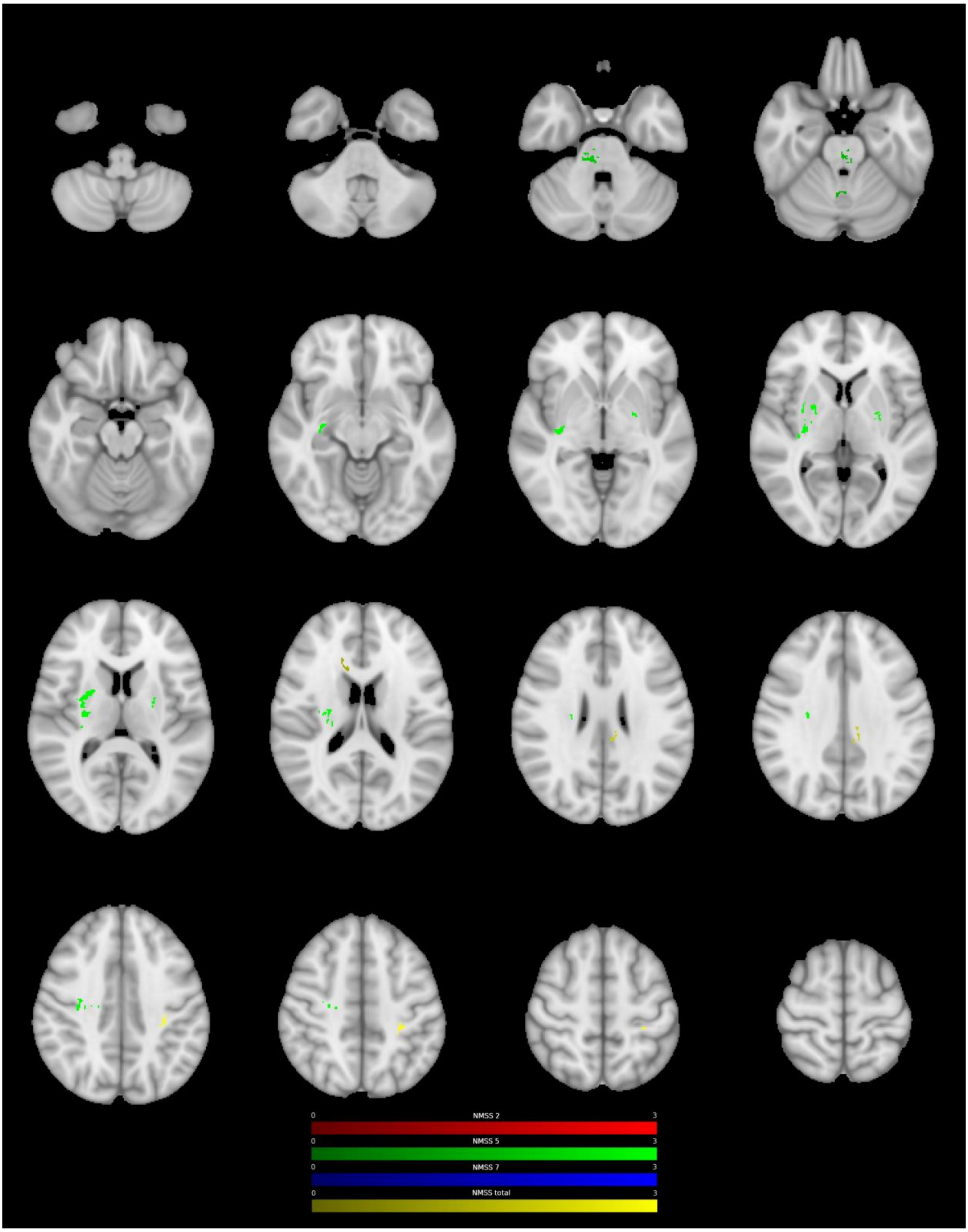

Supplementary Figure 4. Clusters with a negative association between PD patients' NDI-values and postoperative change in NMSS-T (yellow), Domain 2 (sleep/fatigue, red), Domain 5 (attention/memory, green), and Domain 7 (urinary, blue), as revealed by the whole brain analysis. P-Values were corrected for multiple comparisons using a permutation-based approach.

Supplementary Figure 5

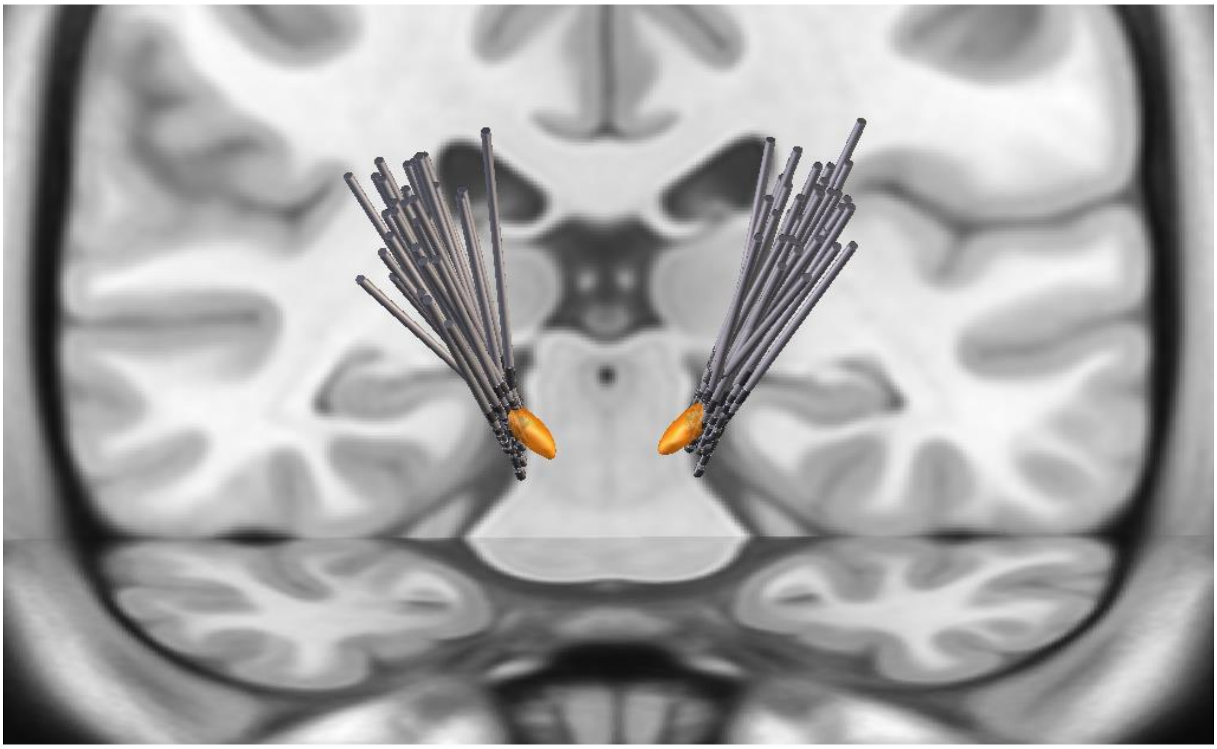

Supplementary Figure 5 Reconstruction of the electrodes for the 37 patients as calculated within the Lead-DBS toolbox.

Supplementary Figure 6

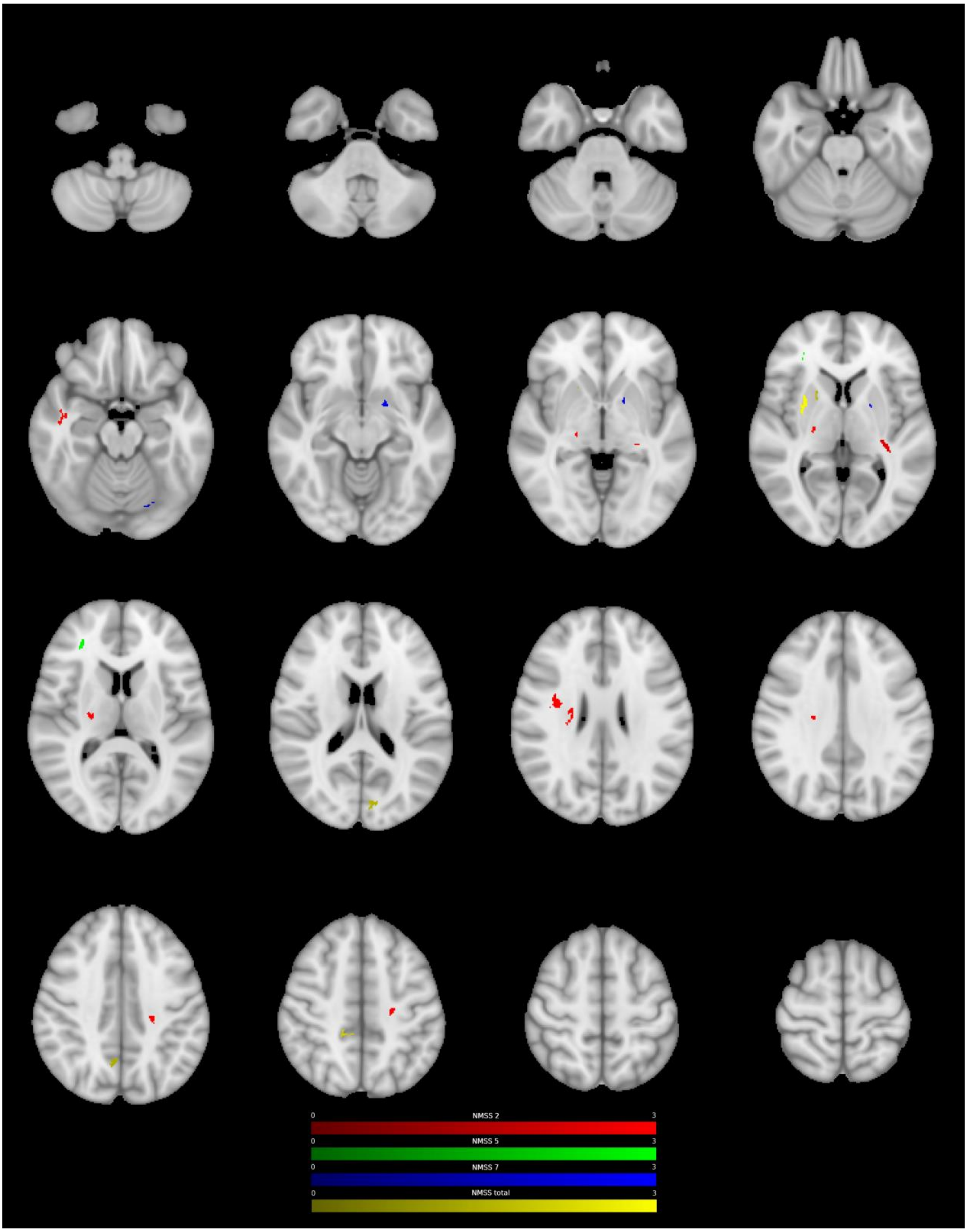

Supplementary Figure 6. Clusters with a positive association between PD patients' ODI-values and postoperative change in NMSS-T (yellow), Domain 2 (sleep/fatigue, red), Domain 5 (attention/memory, green), and Domain 7 (urinary, blue), as revealed by the whole brain analysis, corrected for the average percentage overlap between the VTA and the bilateral STN. P-Values were corrected for multiple comparisons using a permutation-based approach.

Supplementary Figure 7

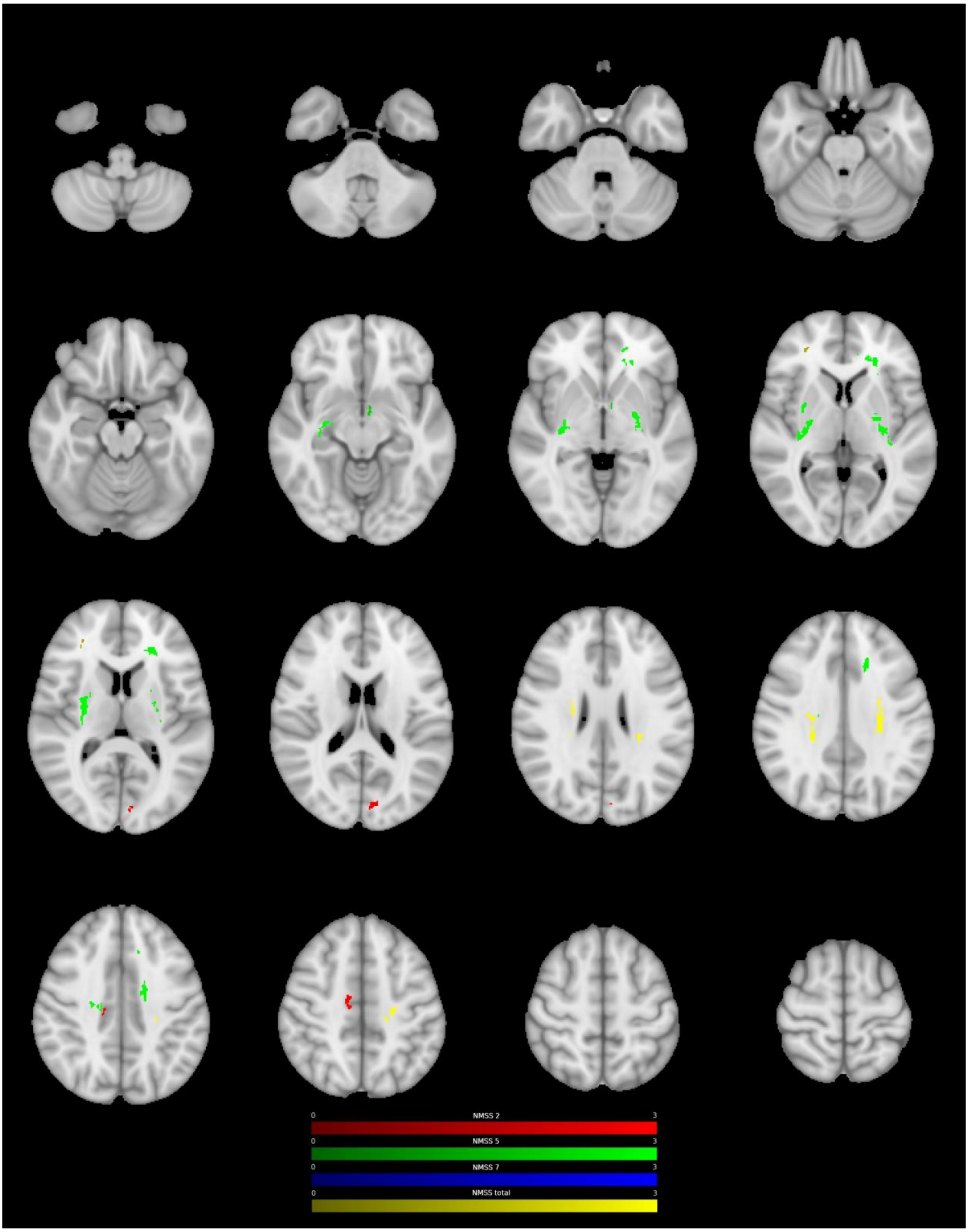

Supplementary Figure 7. Clusters with a negative association between PD patients' ODI-values and postoperative change in NMSS-T (yellow), Domain 2 (sleep/fatigue, red), Domain 5 (attention/memory, green), and Domain 7 (urinary, blue), as revealed by the whole brain analysis, corrected for the average percentage overlap between the VTA and the bilateral STN. P-Values were corrected for multiple comparisons using a permutation-based approach.

Supplementary Figure 8

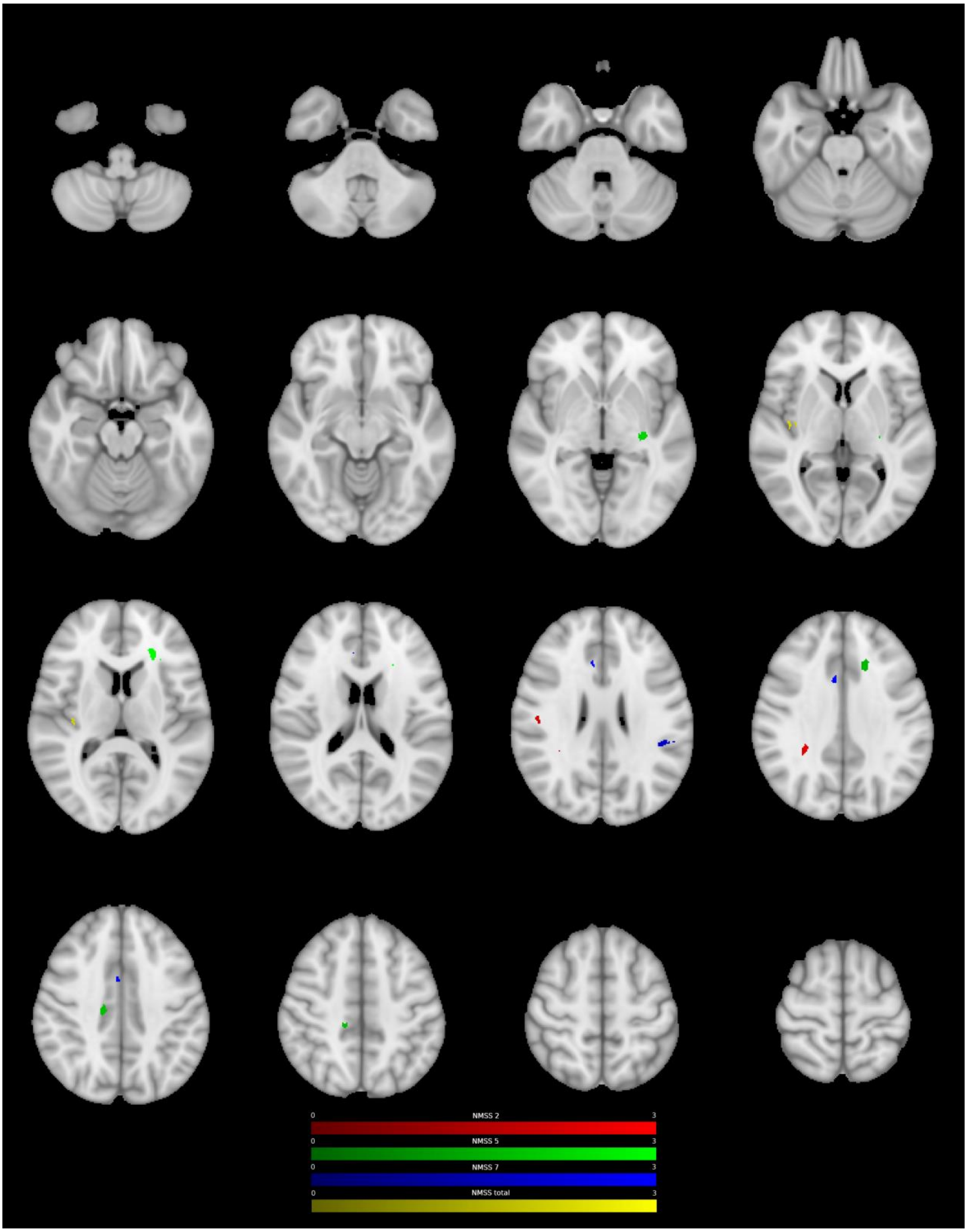

Supplementary Figure 8. Clusters with a positive association between PD patients' FA-values and postoperative change in NMSS-T (yellow), Domain 2 (sleep/fatigue, red), Domain 5 (attention/memory, green), and Domain 7 (urinary, blue), as revealed by the whole brain analysis, corrected for the average percentage overlap between the VTA and the bilateral STN. P-Values were corrected for multiple comparisons using a permutation-based approach.

Supplementary Figure 9

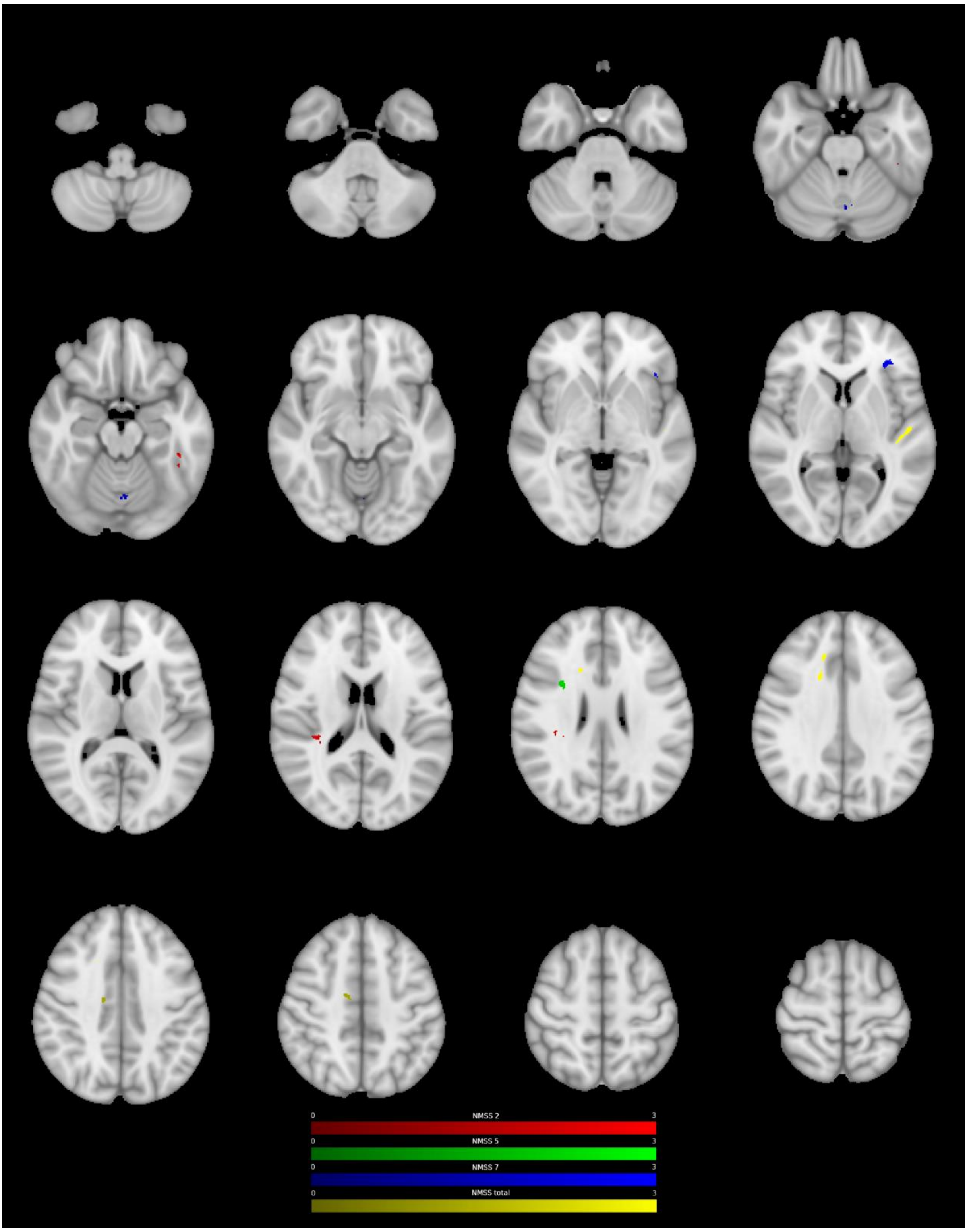

Supplementary Figure 9. Clusters with a negative association between PD patients' FA-values and postoperative change in NMSS-T (yellow), Domain 2 (sleep/fatigue, red), Domain 5 (attention/memory, green), and Domain 7 (urinary, blue), as revealed by the whole brain analysis, corrected for the average percentage overlap between the VTA and the bilateral STN. P-Values were corrected for multiple comparisons using a permutation-based approach.

Supplementary Figure 10

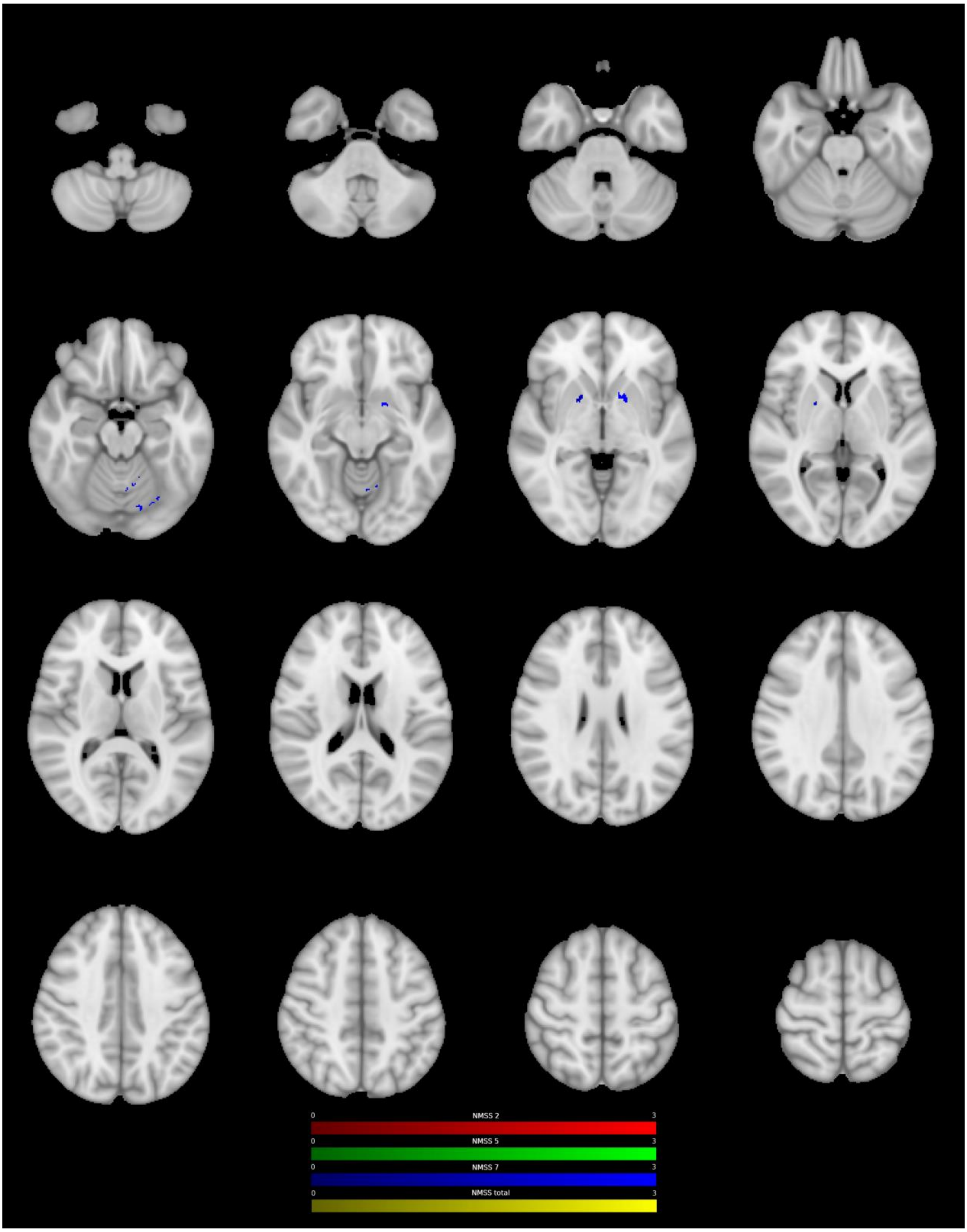

Supplementary Figure 10. Clusters with a positive association between PD patients' NDI-values and postoperative change in NMSS-T (yellow), Domain 2 (sleep/fatigue, red), Domain 5 (attention/memory, green), and Domain 7 (urinary, blue), as revealed by the whole brain analysis, corrected for the average percentage overlap between the VTA and the bilateral STN. P-Values were corrected for multiple comparisons using a permutation-based approach.

Supplementary Figure 11

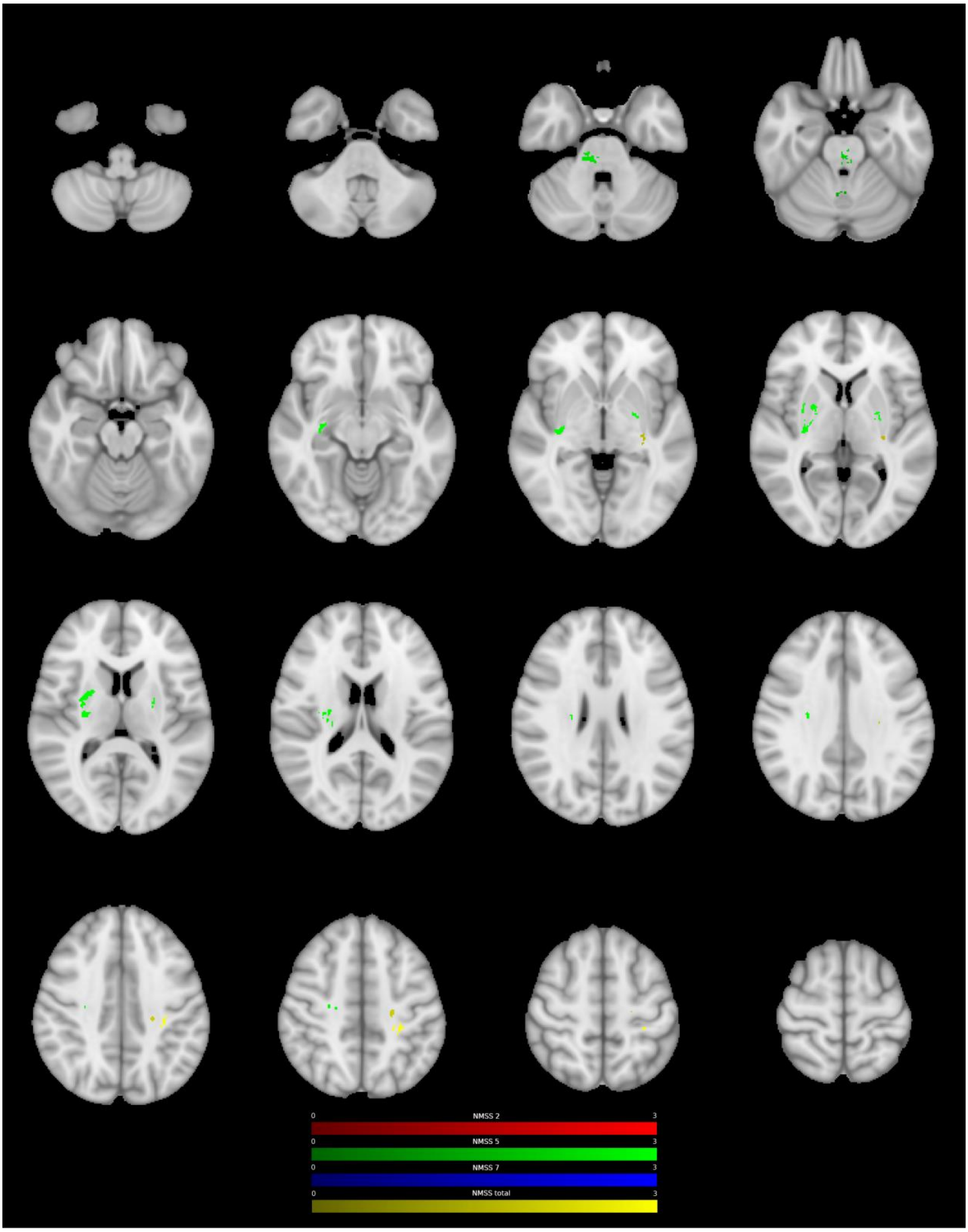

Supplementary Figure 11. Clusters with a negative association between PD patients' NDI-values and postoperative change in NMSS-T (yellow), Domain 2 (sleep/fatigue, red), Domain 5 (attention/memory, green), and Domain 7 (urinary, blue), as revealed by the whole brain analysis, corrected for the average percentage overlap between the VTA and the bilateral STN. P-Values were corrected for multiple comparisons using a permutation-based approach.
