# Supplementary material for: Microstructure predicts non-motor outcomes following deep brain stimulation in Parkinson’s disease
Source: NPJ Parkinsons Dis. 2024 May 18;10:104. doi: 10.1038/s41531-024-00717-y (PMC11102428; doi:10.1038/s41531-024-00717-y)
Supplement: Supplementary file 2 — Reporting Summary [file 41531_2024_717_MOESM2_ESM.pdf]

nature portfolio

Corresponding author(s): Philipp Loehrer

Last updated by author(s): Jan 29, 2024

Reporting Summary

Nature Portfolio wishes to improve the reproducibility of the work that we publish. This form provides structure for consistency and transparency in reporting. For further information on Nature Portfolio policies, see our Editorial Policies and the Editorial Policy Checklist.

Statistics

For all statistical analyses, confirm that the following items are present in the figure legend, table legend, main text, or Methods section.

n/a	Confirmed	
☐	☒	The exact sample size (*n*) for each experimental group/condition, given as a discrete number and unit of measurement
☐	☒	A statement on whether measurements were taken from distinct samples or whether the same sample was measured repeatedly
☐	☒	The statistical test(s) used AND whether they are one- or two-sided<br>*Only common tests should be described solely by name; describe more complex techniques in the Methods section.*
☒	☐	A description of all covariates tested
☐	☒	A description of any assumptions or corrections, such as tests of normality and adjustment for multiple comparisons
☐	☒	A full description of the statistical parameters including central tendency (e.g. means) or other basic estimates (e.g. regression coefficient) AND variation (e.g. standard deviation) or associated estimates of uncertainty (e.g. confidence intervals)
☐	☒	For null hypothesis testing, the test statistic (e.g. *F*, *t*, *r*) with confidence intervals, effect sizes, degrees of freedom and *P* value noted<br>*Give P values as exact values whenever suitable.*
☒	☐	For Bayesian analysis, information on the choice of priors and Markov chain Monte Carlo settings
☒	☐	For hierarchical and complex designs, identification of the appropriate level for tests and full reporting of outcomes
☐	☒	Estimates of effect sizes (e.g. Cohen's *d*, Pearson's *r*), indicating how they were calculated

Our web collection on statistics for biologists contains articles on many of the points above.

Software and code

Policy information about availability of computer code

Data collection	No software was used for collection of clinical data.
Data analysis	All tools used to analyse MRI data are based on FreeSurfer Version 7.1 (http://surfer.nmr.mgh.harvard.edu/) and FSL 6.0.5.2 (http://www.fmrib.ox.ac.uk/fsl) packages, which are freely available. Python program code for the analysis of NODDI-DTI is available from https://github.com/dicemt/DTI-NODDI.

For manuscripts utilizing custom algorithms or software that are central to the research but not yet described in published literature, software must be made available to editors and reviewers. We strongly encourage code deposition in a community repository (e.g. GitHub). See the Nature Portfolio guidelines for submitting code & software for further information.

Data

Policy information about availability of data

All manuscripts must include a data availability statement. This statement should provide the following information, where applicable:

- Accession codes, unique identifiers, or web links for publicly available datasets
- A description of any restrictions on data availability
- For clinical datasets or third party data, please ensure that the statement adheres to our policy

The data supporting this study’s findings are available on request from the corresponding author (PAL). The data are not publicly available due to privacy or ethical restrictions.

Research involving human participants, their data, or biological material

Policy information about studies with human participants or human data. See also policy information about sex, gender (identity/presentation), and sexual orientation and race, ethnicity and racism.

Reporting on sex and gender	In the present study, sex was determined based on self-reporting. As this is an ongoing open-label study recruiting patients that undergo deep brain stimulation of the subthalamic nucleus for Parkinson's disease, neither sex nor gender were considered in the study design. Overall, 9 female and 28 male patients were assessed in this analysis.
Reporting on race, ethnicity, or other socially relevant groupings	We did not obtain information on race, ethnicity or other socially constructed categories.
Population characteristics	There were no covariate-dependent population statistics like genotype used in this study.
Recruitment	Participants were recruited via the University Hospital Marburg (Department of Neurology).
Ethics oversight	The study was approved by the local ethics committee (study-number: 155/17) and carried out following the Declaration of Helsinki.

Note that full information on the approval of the study protocol must also be provided in the manuscript.

Field-specific reporting

Please select the one below that is the best fit for your research. If you are not sure, read the appropriate sections before making your selection.

☒ Life sciences ☐ Behavioural & social sciences ☐ Ecological, evolutionary & environmental sciences

For a reference copy of the document with all sections, see nature.com/documents/nr-reporting-summary-flat.pdf

Life sciences study design

All studies must disclose on these points even when the disclosure is negative.

Sample size	Based on the general trend in the field of microstrucutral and DTI analysis as well as our experience, a sample size of 37 subjects should give meaningful and reasonable results (cf. 10.1002/hbm.23628 or doi: 10.3389/fneur.2020.531993 for recent publications). We used data from 37 subjects for the final analysis. Details are given in the Methods section.
Data exclusions	No data was excluded for final analysis of the association between metrics of microstructure and NMSS-T (and subscore) as well as AES values.
Replication	We repeated the statistical voxelwise analysis of image data for the following metrics: FA, ODI, and NDI. Results yielded several overlapping clusters.
Randomization	Randomization was not relevant for this trial as we investigated the association between metrics of microstructure with postoperative non-motor outcomes.
Blinding	No pharmacological or comparable intervention took place. Furthermore, de-identified data was used during analysis steps. Therefore, blinding was not relevant for this study.

Reporting for specific materials, systems and methods

We require information from authors about some types of materials, experimental systems and methods used in many studies. Here, indicate whether each material, system or method listed is relevant to your study. If you are not sure if a list item applies to your research, read the appropriate section before selecting a response.

Materials & experimental systems

n/a	Involved in the study	
☒	☐	Antibodies
☒	☐	Eukaryotic cell lines
☒	☐	Palaeontology and archaeology
☒	☐	Animals and other organisms
☒	☐	Clinical data
☒	☐	Dual use research of concern
☒	☐	Plants

Methods

n/a	Involved in the study	
☒	☐	ChIP-seq
☒	☐	Flow cytometry
☐	☒	MRI-based neuroimaging

Plants

Seed stocks	-
Novel plant genotypes	-
Authentication	-

Magnetic resonance imaging

Experimental design

Design type	Structural MRI, resting state.
Design specifications	NA
Behavioral performance measures	NA

Acquisition

Imaging type(s)	structural
Field strength	3T
Sequence & imaging parameters	Please see 'MRI Data Acquisition and Processing' in the supplementary materials
Area of acquisition	whole brain

Diffusion MRI ☒ Used ☐ Not used

Parameters	42 diffusion encoding gradients, three intermittent non-weighted b0 images (b = 0 s/mm²), high b-value b = 1000 s/mm², single shell with cardiac gating

Preprocessing

Preprocessing software	DTI data were processed using FMRIB Software Library (FSL) 6.0.5.2 (https://fsl.fmrib.ox.ac.uk/fsl).<br>The processing of T1-weighted scans was performed within the FreeSurfer image analysis suite 7.1.1 (http://surfer.nmr.mgh.harvard.edu)
Normalization	Linear and non-linear transformation to the Montreal Neurological Institute and Hospital data space (MNI152).
Normalization template	Montreal Neurological Institute and Hospital data space (MNI152)
Noise and artifact removal	All images were investigated to be free of subject motion or ghosting and high frequency or wrap-around artefacts at the time of image acquisition.<br>The processing of T1-weighted scans included skull stripping, automated Talairach transformation, cortical and subcortical segmentation, intensity normalisation, tessellation of the grey/white matter boundary, automated topology correction, and surface deformation following intensity gradients.<br>To correct for eddy-current distortions and involuntary movements, raw DTI volumes were linearly registered and resampled to the first b0 volume.
Volume censoring	NA

Statistical modeling & inference

Model type and settings	Generalized linear modelling.
Effect(s) tested	Associations between metrics of brain microstructure and non-motor outcomes were assessed using a generalized linear model.

Specify type of analysis: ☒ Whole brain ☐ ROI-based ☐ Both

Statistic type for inference

(See Eklund et al. 2016)

voxel-wise

Correction

A permutation-based approach based on the Analysis of Functional NeuroImages (AFNI) null-z simulator was used to corrected for multiple comparisons employing 12,000 simulations under the null hypothesis.

Models & analysis

n/a	Involved in the study
☒	☐ Functional and/or effective connectivity
☒	☐ Graph analysis
☒	☐ Multivariate modeling or predictive analysis
